# ZNF512B binds RBBP4 via a variant NuRD interaction motif and aggregates chromatin in a NuRD complex-independent manner

**DOI:** 10.1093/nar/gkae926

**Published:** 2024-10-26

**Authors:** Tim Marius Wunderlich, Chandrika Deshpande, Lena W Paasche, Tobias Friedrich, Felix Diegmüller, Elias Haddad, Carlotta Kreienbaum, Haniya Naseer, Sophie E Stebel, Nadine Daus, Jörg Leers, Jie Lan, Van Tuan Trinh, Olalla Vázquez, Falk Butter, Marek Bartkuhn, Joel P Mackay, Sandra B Hake

**Affiliations:** Institute for Genetics, Justus-Liebig University Giessen, Heinrich-Buff-Ring 58-62, 35392 Giessen, Germany; School of Life and Environmental Sciences, Butlin Ave, University of Sydney, Darlington, New South Wales 2006, Australia; Institute for Genetics, Justus-Liebig University Giessen, Heinrich-Buff-Ring 58-62, 35392 Giessen, Germany; Biomedical Informatics and Systems Medicine Science Unit for Basic and Clinical Medicine, Justus-Liebig University Giessen, Aulweg 128, 35392 Giessen, Germany; Institute for Genetics, Justus-Liebig University Giessen, Heinrich-Buff-Ring 58-62, 35392 Giessen, Germany; Institute for Genetics, Justus-Liebig University Giessen, Heinrich-Buff-Ring 58-62, 35392 Giessen, Germany; Institute for Genetics, Justus-Liebig University Giessen, Heinrich-Buff-Ring 58-62, 35392 Giessen, Germany; Institute for Genetics, Justus-Liebig University Giessen, Heinrich-Buff-Ring 58-62, 35392 Giessen, Germany; Institute for Genetics, Justus-Liebig University Giessen, Heinrich-Buff-Ring 58-62, 35392 Giessen, Germany; Institute for Genetics, Justus-Liebig University Giessen, Heinrich-Buff-Ring 58-62, 35392 Giessen, Germany; Institute for Genetics, Justus-Liebig University Giessen, Heinrich-Buff-Ring 58-62, 35392 Giessen, Germany; Institute for Genetics, Justus-Liebig University Giessen, Heinrich-Buff-Ring 58-62, 35392 Giessen, Germany; Department of Chemistry, Philipps University Marburg, Hans-Meerwein-Straße 4, 35043 Marburg, Germany; Department of Chemistry, Philipps University Marburg, Hans-Meerwein-Straße 4, 35043 Marburg, Germany; Center for Synthetic Microbiology, Philipps University Marburg, Karl-von-Frisch-Str. 14, 35043 Marburg, Germany; Institute of Molecular Biology (IMB), Ackermannweg 4, 55128 Mainz, Germany; Institute of Molecular Virology and Cell Biology, Friedrich-Loeffler-Institute, Federal Research Institute for Animal Health, Südufer 10, 17493 Greifswald, Germany; Biomedical Informatics and Systems Medicine Science Unit for Basic and Clinical Medicine, Justus-Liebig University Giessen, Aulweg 128, 35392 Giessen, Germany; School of Life and Environmental Sciences, Butlin Ave, University of Sydney, Darlington, New South Wales 2006, Australia; Institute for Genetics, Justus-Liebig University Giessen, Heinrich-Buff-Ring 58-62, 35392 Giessen, Germany

## Abstract

The evolutionarily conserved histone variant H2A.Z plays a crucial role in various DNA-based processes, but the mechanisms underlying its activity are not completely understood. Recently, we identified the zinc finger (ZF) protein ZNF512B as a protein associated with H2A.Z, HMG20A and PWWP2A. Here, we report that high levels of ZNF512B expression lead to nuclear protein and chromatin aggregation foci that form in a manner that is dependent on the ZF domains of ZNF512B. Notably, we demonstrate ZNF512B binding to the nucleosome remodeling and deacetylase (NuRD) complex. We discover a conserved amino acid sequence within ZNF512B that resembles the NuRD-interaction motif (NIM) previously identified in FOG-1 and other transcriptional regulators. By solving the crystal structure of this motif bound to the NuRD component RBBP4 and by applying several biochemical and biophysical assays, we demonstrate that this internal NIM is both necessary and sufficient for robust and high-affinity NuRD binding. Transcriptome analyses and reporter assays identify ZNF512B as a repressor of gene expression that can act in both NuRD-dependent and -independent ways. Our study might have implications for diseases in which ZNF512B expression is deregulated, such as cancer and neurodegenerative diseases, and hints at the existence of more proteins as potential NuRD interactors.

## Graphical Abstract

**Graphical Abstract:**
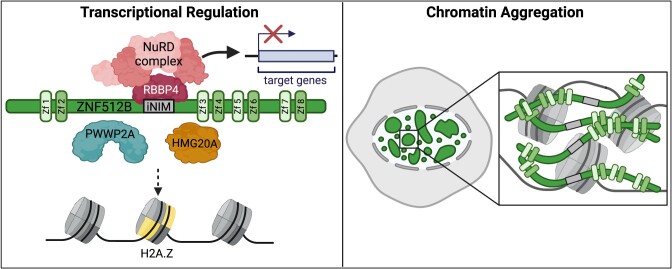

## Introduction

Zinc finger (ZF) proteins are the most abundant protein family in eukaryotes and are characterized by the coordination of one or more zinc ions through a combination of cysteine and histidine residues (1,2). ZFs exhibit the ability to bind a variety of molecules, including DNA, RNA, lipids and proteins (3–6), playing pivotal roles in numerous DNA-based processes such as development, differentiation, metabolism signal transduction and immune responses (1). Many ZF proteins have been shown to act as transcription factors (TFs), often by facilitating the recruitment of other, enzymatically active proteins to specific DNA sequences. As a consequence of this recruitment, the structure of chromatin (7), the regulatory packaged form of the genetic material in eukaryotes, undergoes modifications that impact its functionality. The smallest unit of chromatin is the nucleosome, an octameric complex comprising two of each of the four core histones H2A, H2B, H3 and H4 wrapped by approximately 150 bp of DNA (8). Various interconnected mechanisms have evolved to ensure the local and/or global control of nucleosome positions and DNA accessibility, including the addition of chemical groups to DNA or histone proteins, ATP-dependent remodeling and incorporation of specialized histone variants (9).

One extensively studied histone variant is the conserved and essential (in higher eukaryotes) histone variant H2A.Z (10). Its deposition into genomic regulatory regions, such as promoters and enhancers, governs transcription, genome stability and cell cycle progression. Additionally, it plays a crucial role during stem cell differentiation and early development (11–13). H2A.Z is believed to influence these processes amongst other things through the binding and recruitment of various chromatin-modifying complexes (14).

In previous studies, we identified proteins enriched on H2A.Z-containing nucleosomes compared to those harbouring the replication-dependent histone H2A (15,16). We discovered several chromatin-modifying complexes and functionally understudied proteins, such as PWWP Domain-Containing Protein 2A (PWWP2A), High Mobility Group Protein 20A (HMG20A) and ZF Protein 512B (ZNF512B). We demonstrated that PWWP2A is a direct H2A.Z nucleosome interactor required for head development. Furthermore, it binds to an Metastasis-associated protein 1 (MTA1)-specific core nucleosome remodeling and deacetylase (NuRD) complex that we termed M1HR (17,18). This M1HR sub-complex lacks its remodeling Chromodomain-helicase-DNA-binding protein (CHD)/Methyl-CpG-binding domain protein (MBD)/GATA zinc finger domain-containing protein (GATA) module due to competition between PWWP2A and MBD proteins for binding to MTA1 (19).

In addition to M1HR, the PHD Finger 14 (PHF14), Retinoic Acid Induced 1 (RAI1), Transcription Factor 20 (TCF20) and HMG20A proteins have been consistently identified in both H2A.Z.1/2.1 and PWWP2A mass-spectrometry-based interactomes (15–17). In a recent follow-up study, we discovered that HMG20A is enriched at regulatory regions and controls important early head and heart transcription programs (20). In contrast to H2A.Z and PWWP2A, HMG20A was able to pull down all components of the BHC/CoREST and NuRD repressive complexes, the latter including both remodeling (CHD) and deacetylase (HDAC) subunits.

Within this and other quantitative mass-spectrometry screens, we also identified ZNF512B to be associated with H2A.Z, PWWP2A and HMG20A. ZNF512B, previously named GAM/ZFp (21), is a vertebrate-specific ZF protein (22). Few studies exist on ZNF512B’s function. The studies that are available demonstrate its role in gene regulation and cell homeostasis (22), indicate a possible association with NuRD in a mass-spectrometry based screen (23) and suggest a putative, yet unclear, role in the neurodegenerative disease amyotrophic lateral sclerosis (ALS) (24–26).

Here, we demonstrate that ZNF512B, when overexpressed, can induce severe chromatin compaction in a ZF-dependent manner, likely because of its DNA-binding and oligomerization abilities. We identified a conserved amino acid sequence within ZNF512B, resembling the functional NuRD-interaction motif (NIM) first detected in the TF Friend of GATA protein 1 (FOG-1), also known as FOG-1-like motif (27). Using a lysine-to-alanine NIM mutant, we demonstrate that this putative FOG-1-like motif in ZNF512B is a functional protein–protein interaction domain, both necessary and sufficient for NuRD binding. We show that ZNF512B acts as transcriptional repressor, in both NuRD-dependent and NuRD-independent ways, and that its overexpression or depletion leads to a deregulation of gene expression.

Moreover, the crystal structure of the ZNF512B NIM bound to the NuRD component Retinoblastoma-binding protein 4 (RBBP4) reveals a binding mode that is overall conserved with other RBBP4 complexes, although we also observed highly interesting distinct differences. A mutagenesis screen of this motif confirms the observed interactions and defines a variant internal NuRD binding sequence. Comparison of NIMs across many characterized NuRD-interacting proteins implies the presence of two different consensus sequences, which appear to correlate with the location of the motif in the protein sequence: (i) an N-terminal RRKQxxP and (ii) an internally located RK(x)xxPxK/R motif. Finally, we demonstrate that the ZNF512B NIM has a several times higher binding affinity for RBBP4 than the N-terminal NIM of FOG-1.

In summary, ZNF512B influences chromatin architecture, when present in elevated levels, interacts directly with RBBP4 of the NuRD complex using an internal binding motif and represses transcription.

## Materials and methods

### Cell culture and transfections

HeLa Kyoto (HeLaK), U2OS and HEK293T cells were cultured in Dulbecco’s modified Eagle medium (DMEM, Gibco), HCT116 cells were cultured in McCoy’s 5A modified Medium, both supplemented with 10% fetal calf serum (FCS, Gibco) and 1% penicillin/streptomycin (37°C, 5% CO_2_). Sf9 cells were cultured in Sf-900TM II SFM medium (Gibco) and maintained at 27°C and 90 rpm. Cells were routinely tested via PCR for mycoplasma contamination. Transfections of HeLaK, U2OS, HCT116 and Sf9 cells were performed using FuGENE® HD Transfection Reagent (Promega) according to the manufacturer’s instructions (Promega). HEK293T cells were transfected using the calcium phosphate method as previously described (28). Unless stated otherwise, cells were harvested by trypsinization for various assays 48 h after transfection.

### Cloning of ZNF512B and its mutants

To generate human ZNF512B (mutant) plasmids, total RNA of HeLaK cells was purified using RNeasy-Kit (QIAGEN) and reverse transcribed using Transcriptor First Strand cDNA Synthesis Kit (Roche). cDNA was amplified by Q5® High-Fidelity DNA Polymerase (New England Biolabs) and cloned into pIRESneo-GFP (29), pEGFP-N2, p3xFLAG-CMV-10, pFastBac1 (Invitrogen), pAB-Gal94 (30) and pTetLS (gift from Prof. Lienhard Schmitz, JLU Giessen) vectors. Point mutation constructs were generated via site-directed mutagenesis (31).

### Primers and oligos

All primers and oligos are listed in the Supplementary Data.

### Generation of tet-inducible GFP/GFP-ZNF512B cell lines

HeLaK cells were transfected with pTetLS-eGFP or pTetLS-eGFP-ZNF512B plasmids together with px330-AAVS1-T2-CRISPR (gift from Prof. Lienhard Schmitz, JLU Giessen) coding for Cas9 and a guide RNA targeting the AAVS1 safe harbor locus. Two days after transfection, 10 μg/ml Blasticidin S was added to the cells to begin selection. Expression of GFP or GFP-ZNF512B was induced with 1 μg/ml doxycycline.

### Immunofluorescence microscopy

Immunofluorescence (IF) staining and microscopy was performed as previously described (32). Briefly, 1 × 10^5^ adherent cells expressing GFP, GFP-ZNF512B or its deletions/mutants were seeded on coverslips in 24-well plates and cultured overnight. The next day, cells were washed two times with PBS and fixed for 15 min in PBS containing 1% formaldehyde. After washing, cells were permeabilized and blocked with PBS containing 0.1% Triton™ X-100 (PBS-T) and 1% bovine serum albumin (BSA) for 20 min. Cells were incubated stepwise with primary and then secondary antibodies in PBS-T + 1% BSA for 30 min with three wash-steps using PBS-T in between. After three final PBS-T wash-steps, DNA was stained with 10 μg/ml Hoechst solution for 3 min. After washing with H_2_O, coverslips with cells were then mounted in Fluoromount-G® mounting medium (SouthernBiotech) on microscope slides. Images were acquired using an Axio Observer.Z1 inverted microscope (Carl Zeiss, Oberkochen, Germany) with an Axiocam 506 mono camera system. Image processing was performed with Zeiss Zen (version 3.1, blue.edition). Intensity profiles were created using ImageJ (version 1.53t). Details on applied antibodies are listed in the Supplementary Data.

### *I* *n situ* staining for β-galactosidase activity

A total of 1 × 10^5^ HeLaK cells expressing GFP or GFP-ZNF512B or treated with doxorubicin (100 nM, 2 days, Fisher Scientific) were seeded on coverslips in 24-well plates. *In situ* staining was done as described (33). In brief, after washing with PBS and fixing with 3.7% formaldehyde, cells were incubated overnight at 37°C in freshly prepared staining buffer (PBS pH 6.0, 2 mM MgCl_2_, 5 mM K_3_Fe[CN]_6_, 5 mM K_4_Fe[CN]_6_, 1 mg/ml X-Gal). After washing with H_2_O, coverslips with cells were mounted and imaged as described for IF microscopy.

### TUNEL assay

TUNEL assays were performed using the One-Step TUNEL In Situ Apoptosis Kit (Elabscience) following the manufacturer’s instructions. In brief, HeLaK cells expressing GFP or GFP-ZNF512B were fixed and permeabilized as described for IF microscopy. For the positive control, coverslips were incubated with DNase I buffer for 5 min at RT and afterwards with DNase I working solution for 30 min at 37°C. For the negative control, coverslips were incubated with DNase I buffer for 5 min at RT and afterward for 30 min at 37°C. Coverslips for all samples were then incubated with TdT equilibration buffer for 30 min at 37°C and subsequently with labelling working solution for 60 min at 37°C in a humidified chamber in the dark. Finally, coverslips were incubated with 4′,6-diamidino-2-phenylindole (DAPI) working solution for 5 min at RT in the dark and mounted on microscope slides as described for IF microscopy.

### Antibodies

All antibodies are listed in the Supplementary Data.

### Electrophoretic mobility shift assays (EMSAs)

Expression and purification of recombinant His-ZNF512B protein was done as described (32). Briefly, pFastBac1 vectors were transformed into DH10Bac bacteria and viruses were generated according to the Bac-to-Bac™ Baculovirus Expression System Protocol (Invitrogen). Extracts were prepared 3 days after infection by washing the cells twice with ice cold PBS and incubation on ice for 10 min in hypotonic buffer (10 mM HEPES-KOH pH 7.9, 1.5 mM MgCl_2_, 10 mM KCl). After brief vortexing and centrifugation, the pellet (nuclei) was incubated for 20 min in hypertonic buffer (20 mM HEPES-KOH pH 7.9, 25% glycerol, 420 mM NaCl, 1.5 mM MgCl_2_, 0.2 mM EDTA). After centrifugation for 10 min at 16 000 × *g* the supernatant was stored at −20°C or directly used for electrophoretic mobility shift assays (EMSAs). Alternatively, the supernatant was purified by FPLC using the Äkta Start (Cytiva) via a His-Trap column (71502768) by standard procedures outlined in the instructions of the manufacturer.

EMSAs were performed as originally described (34) with the exception that 300 ng of salmon sperm DNA per reaction were used as an unspecific competitor. Cy5 end-labeled unmethylated or Cy3 end-labeled methylated double-stranded oligos (in which all eight Cytosines in a CpG context were methylated) were used as EMSA probes; the sequence was derived from the mouse H19 promoter (for sequence details see Supplementary Data).

### DNase immunoprecipitation (DNase-IP) and peptide competition

Preparation of DNase-digested cell extracts and immunoprecipitation was performed using GFP-Trap® Magnetic Particles M-270 (Chromotek) following the manufacturer’s protocol. In brief, cells were incubated for 30 min on ice with RIPA buffer (10 mM Tris/Cl pH 7.5, 150 mM NaCl, 0.5 mM EDTA, 0.1% SDS, 1% Triton™ X-100, 1% deoxycholate) supplemented with DNase I (75 Kunitz U/ml, Thermo Fisher Scientific) and MgCl_2_ (2.5 mM). After centrifugation at 17 000 *× g* for 10 min at 4°C, lysate was transferred to a fresh tube and mixed with Dilution buffer (10 mM Tris/Cl pH 7.5, 150 mM NaCl, 0.5 mM EDTA). Lysate was then incubated with GFP-Trap® Magnetic Particles M-270 rotating for 1 h at 4°C. For peptide competition, lysate was incubated with peptide solution rotating for 1 h at 4°C before incubation with beads. After incubation, beads were washed three times with Wash buffer (10 mM Tris/Cl pH 7.5, 150 mM NaCl, 0.05% Nonidet™ *P*-40 Substitute, 0.5 mM EDTA), and precipitated proteins were eluted by boiling beads for 5 min in 2× SDS-sample buffer. Eluates were compared to input material via semi-dry western blotting. Details on applied antibodies and peptides are listed in the Supplementary Data.

### Fluorescence polarization

Expression of recombinant RBBP4 protein was done as described (32). RBBP4 was purified by FPLC using the Äkta Start (Cytiva) via a His-Trap column (71502768) and subsequently a HiTRAP Q column (29051325) by standard procedures outlined in the instructions of the manufacturer. To determine the dissociation constant (*k*_D_), serial dilutions of RBBP4 protein (10–0 μM, 25 μl) in 50 mM Tris/Cl pH 7.5, 150 mM NaCl and 0.02% Triton™ X-100 were added to 25 μl of the fluorescently tagged tracer peptide for a finale volume of 50 μl with 1 nM tracer. The 384-well plate (GBO 781 900) was measured (Tecan Spark 20M) after 60 min and the *k*_D_ values were calculated by converting the mP values into their corresponding anisotropy (*A*) values following the equation:


\begin{equation*}A = \frac{{2P}}{{3 - P}}\end{equation*}

Hereby, *A* = anisotropy and *P* = polarization. These values were plotted versus the respective concentrations of the protein with a non-linear regression according to the following equation:


\begin{equation*}A = A_f + \left( A_b - A_f \right)\frac{(L_{\rm T} + k_{\rm D} + R_{\rm T} - \sqrt {\left( L_{\rm T} - k_{\rm D} - R_{\rm T} \right)^2 - 4L_{\rm T}R_{\rm T}}} {2L_{\rm T}}\end{equation*}

Hereby, *A* is the experimental anisotropy, *A_f_* is the anisotropy for the free ligand, *A_b_* is the anisotropy for the fully bound ligand, *L*_T_ is the total added concentration of ligand, *k*_D_ is the equilibrium dissociation constant and *R*_T_ is the total receptor concentration. For the competitive binding experiments RBBP4 protein was preincubated with tracer peptide in 50 mM Tris-HCl pH 7.5, 150 mM NaCl and 0.02% Triton™ X-100 for 60 min to form the protein-tracer complex. To 25 μl of this protein-tracer complex, 25 μl of the corresponding peptide serial dilutions (1.0–0.0 mM) were added, with a final concentration of 1 nM tracer, 40 nM protein. After 60 min the polarization values were read and plotted against the logarithmic peptide concentration to determine the inhibitory concentration (IC_50_) by fitting the dose-response to four parameter logistic (4-PL) curves in GraphPad Prism 6. Inhibition constant (*k_i_*) values were calculated using the following equation described previously by Wang *et al.* and the standard deviation was calculated for each *k_i_* value (35).


\begin{equation*}{{k}_i} = \frac{{{{{\left[ I \right]}}_{50}}}}{{\frac{{{{{[L]}}_{50}}}}{{{{k}_D}}} + \frac{{{{{\left[ P \right]}}_0}}}{{{{k}_D}}} + 1}}\end{equation*}

Hereby, *k_i_* is the competitive inhibition constant, [*I*]_50_ is the concentration of free inhibitor at 50% inhibition, [*L*]_50_ is the concentration of free labelled ligand at 50% inhibition, and [*P*]_0_ is the concentration of free protein at 0% inhibition.

### Peptides

All peptides are listed in Supplementary Figure S6A–F.

### MNase immunoprecipitation (MNase-IP) and MS sample preparation

Preparation of Micrococcal nuclease (MNase)-digested nuclear extracts was performed as previously described (15–17,32). Briefly, nuclei were isolated by incubation with PBS containing 0.3% Triton™ X-100 for 10 min at 4°C and washed in PBS, before being resolved in 500 μl freshly prepared Ex100 buffer (10 mM HEPES pH 7.6, 100 mM NaCl, 1.5 mM MgCl_2_, 0.5 mM EGTA, 10% glycerol, 10 mM β-glycerol phosphate, 1 mM Dithiothreitol (DTT), 2 mM CaCl_2_). Chromatin was then digested with 1.5 U MNase (sufficient for 4 × 10^7^ cells, Sigma-Aldrich) for 20 min at 26°C. Digestion was stopped by adding 10 mM EGTA and transferring the sample to 4°C. After centrifugation for 30 min at 13 000 rpm at 4°C, supernatant containing mostly mononucleosomes was transferred to a fresh reaction tube. To assess integrity of mononucleosomes, DNA was isolated from 25 μl of sample using the QIAquick PCR Purification Kit (QIAGEN) and subjected to agarose gel electrophoresis. For IP experiments, MNase-digested nuclear extracts were incubated overnight with GFP-Trap® Magnetic Particles M-270 (Chromotek) rotating at 4°C. Beads were washed twice with 1 ml Washing Buffer I (10 mM Tris/Cl pH 7.5, 150 mM NaCl, 0.1% Nonidet™ *P*-40 substitute and twice with 1 ml Washing Buffer II (10 mM Tris/Cl pH 7.5, 150 mM NaCl).

For immunoblot analysis, precipitated proteins were eluted by boiling the beads for 5 min in SDS-sample buffer. Eluates were compared to input material via semi-dry Western blotting. Details on applied antibodies are listed in Supplementary Data.

For label-free quantitative mass spectrometry (lf-qMS), precipitated proteins were eluted by incubation of beads with Elution Buffer (2 M urea, 50 mM Tris/Cl pH 7.5, 2 mM DTT, 20 μg/ml Trypsin Gold [Promega]) for 30 min at 37°C shaking at 1400 rpm in the dark. Eluted peptides in the supernatant were transferred into a fresh reaction tube. Peptides remaining bound to beads were alkylated/eluted in Alkylation Buffer (2 M urea, 50 mM Tris/Cl pH 7.5, 10 mM chloroacetamide) for 5 min at 37°C shaking at 1400 rpm in the dark. Both eluates were combined, and peptides were further alkylated and digested overnight at 25°C shaking at 800 rpm in the dark. Trypsin digestion was stopped by adding 1% trifluoroacetic acid (Thermo Fisher Scientific). Peptides were subjected to lf-qMS (see below). Lf-qMS experiments were performed twice (biological replicates) with four technical replicates each.

### Label-free quantitative mass spectrometry (lf-qMS)

#### MS data acquisition

After tryptic digest (see MNase IP), peptides were desalted on StageTips (36) and analysed by nanoflow liquid chromatography on an EASY-nLC 1000 system (Thermo Fisher Scientific) coupled online to a Q Exactive Plus mass spectrometer (Thermo Fisher Scientific). Peptides were separated on a C18-reversed phase capillary (25 cm long, 75 μm inner diameter, packed in-house with ReproSil-Pur C18-AQ 1.9 μm resin, Dr Maisch GmbH) directly mounted on the electrospray ion source. We used a 90 min gradient from 2% to 60% acetonitrile in 0.5% formic acid at a flow of 225 nl/min. The Q Exactive Plus was operated with a Top10 MS/MS spectra acquisition method per MS full scan.

#### MS data analysis

The raw files were processed with MaxQuant (37) (version 1.6.5.0) against a human uniprot database (81194 entries). Carbamidomethylation was set as fixed modification while methionine oxidation and protein *N*-acetylation were considered as variable modifications. The search was performed with an initial mass tolerance of 7 ppm mass accuracy for the precursor ion and 20 ppm for the MS/MS spectra in the HCD fragmentation mode. Search results were processed with MaxQuant and filtered with a false discovery rate of 0.01. The match between run option and the LFQ quantitation were activated. Protein groups marked as reverse, contaminants, only identified by site and <2 peptides (at least 1 unique) were removed. Non-measured values were imputed with a beta distribution at the limit of quantitation. For creating the volcano plots, a *P*-value of a two-tailed Welch’s *t*-test was calculated. Plots were created using the ggplot2 package.

### Structure

#### Peptide synthesis

A peptide comprising residues 419–430 of human ZNF512B (UniProt accession number Q96KM6) was chemically synthesized and purified by ChinaPeptides (Shanghai, China). The peptide was N-terminally acetylated and C-terminally amidated.

#### Expression and purification of recombinant RBBP4

Recombinant expression of full-length RBBP4 (residues 1–425; UniProt accession number Q09028) was carried out as described in (38). Briefly, the pFBDM vector carrying the full length RBBP4 with an N-terminal 6xHis-tag and a thrombin protease cleavage site was used for expression in insect cells. Recombinant baculovirus generation and large-scale expression in Sf9 cells were carried out using the Bac-to-Bac™ (Invitrogen) expression system.

The harvested cells were resuspended on ice in lysis buffer (20 mM Tris/Cl pH 8.0, 150 mM NaCl, 10 mM MgCl_2_, 1 mM β-mercaptoethanol, Complete® EDTA-free protease inhibitor tablet). Cells were lysed by sonication, NP-40 (0.1% v/v) and DNase I (10 μg/ml) were added to the cell lysate and the lysate was then clarified by centrifugation. The supernatant was incubated with nickel-nitrilotriacetic acid beads (Qiagen) for 2 h at 4°C. The beads were washed with lysis buffer containing increasing concentrations of imidazole (2, 20 and 40 mM) and RBBP4 was eluted with 500 mM imidazole. 6xHis-tag was cleaved by dialysing the eluted protein overnight at 4°C in 20 mM Tris/Cl pH 8.0, 150 mM NaCl and 2.5 mM CaCl_2_, together with 500 U of thrombin. RBBP4 was further purified by size-exclusion chromatography, using a HiLoad™ 16/60 Superdex™ 75 pg column (Cytiva) in 20 mM Tris/Cl pH 7.5, 150 mM NaCl and 1 mM DTT. Fractions containing purified RBBP4 were concentrated to 5 mg/ml and stored at −20°C until required.

#### Crystallization and structure determination

Synthetic ZNF512B peptide (residues 419–430) was dissolved in demineralized water at a concentration of 10 mM. Concentrated RBBP4 (5 mg/ml) was mixed with ZNF512B peptide at a molar ratio of 1:2 and incubated on ice for 2 h. Crystallization trials were set up at 4°C in 96-well plates as sitting drops using 0.2 μl of protein solution and 0.1 μl crystallization solution. Single crystals were obtained in 0.1 M MES/imidazole, pH 6.5, 40% v/v ethylene glycol; 20% w/v PEG 8000, 0.1 M mixture of carboxylic acids (Morpheus screen, Molecular Dimensions). The crystals were harvested into a cryoprotectant solution containing 25% glycerol in mother liquor before cryocooling in liquid nitrogen. X-ray diffraction data were collected on MX2 beamline (microfocus) at the Australian Synchrotron at 100 K and a wavelength of 0.9537 Å. The dataset was processed and scaled using XDS (PMID: 20124692) and Scala (PMID: 16369096) respectively, at 2.2 Å resolution. The RBBP4–ZNF512B structure was solved by molecular replacement using PHENIX phaser-MR (PMID: 12393927) with RBBP4–FOG-1 (PDB: 2XU7) as a search model without the FOG-1 or other ligands. The crystal belonged to space group P 1 21 1, with two molecules in the asymmetric unit. Further model building was performed by iterative rounds of manual model building in real space using COOT (39), followed by refinement using REFMAC5 (40) and PHENIX (41). Validation of the structure was carried out with MolProbity (42). Interface analysis was performed using the EBI PISA server (43) and PDBsum (44). Figures were generated using PyMOL (45). The coordinates and structure factors have been deposited in the Protein Data Bank (PDB: 8TX8). Data collection and refinement statistics are outlined in the Supplementary Data.

### Luciferase reporter assay

Per well of a 6-well plate, 1.5 × 10^5^ HEK293T cells were seeded and transfected on the next day using 1 μg of 4xUAS-tk-luc reporter plasmid, 0.5 μg GAL expression vector and 0.5 μg pCMV-lacZ vector for normalization. Cells were directly lysed in well after 72 h with 200 μl Lysis buffer (25 mM Tris/Cl pH 7.5, 8 mM MgCl_2_, 1 mM EDTA, 1% Triton™ X-100, 15% glycerin, 1 mM DTT) for 15 min at RT. Supernatants were used to measure luciferase activity by mixing 100 μl 1:1 with 90 mg/L D-Luciferin/0.8 mM ATP and quantifying emitted light after 5 s incubation with an Orion L plate reader (Titertek-Berthold). Values were normalized to β-galactosidase activity by adding 5 μL supernatant to 900 μl LacZ buffer (50 mM Na_2_HPO_4_, 10 mM KCl, 0.1 mM MgSO_4_, pH 7) and 100 μl 4 g/L OPNG/0.1 M KPO_4_. When a color change of the mixture occurred, absorbance at 420 nm was measured.

### RNA interference (RNAi) mediated knock-down of ZNF512B

For RNAi-mediated knock-down (KD), ON-TARGETplus SMARTpool siRNAs (Horizon Discovery, Cambridge, UK) targeting ZNF512B mRNA or a non-targeting control pool were transfected using DharmaFECT1 transfection reagent (Horizon Discovery, Cambridge, UK) according to the manufacturer's instructions. Briefly, for a 6-well plate 1 × 10^5^ HelaK cells were seeded 24 h before transfection. Ten microliters of 5 μM targeting or non-targeting siRNAs were mixed with 190 μl of reduced serum medium (Opti-MEM™, Gibco). In a separate tube, 4 μl of DharmaFECT1 were mixed with 196 μl Opti-MEM™. Both tubes were incubated for 5 min, before combining and incubating for further 20 min. In the meantime, cells were washed once with PBS followed by the addition of 1.6 ml DMEM supplemented with 10% FCS without antibiotics. The transfection reaction was added dropwise, and cells were cultured for two days before experiments were conducted.

### RNA extraction, RT-qPCR and RNA-seq

RNA extraction was performed as described (46). Briefly, total RNA was extracted using the RNeasy Mini Kit (QIAGEN) with on column DNase digest (RNase-Free DNase Set, QIAGEN) according to the manufacturer’s instructions. One microgram of total RNA was reverse transcribed using the Transcriptor First Strand cDNA Synthesis Kit (Roche) with random hexamer primers according to the manufacturer’s protocol. For subsequent qPCR analysis, technical triplicates of 6 μl cDNA (1:20 dilution), 7.5 μl iTaq™ Universal SYBR® Green Supermix (Bio-Rad) and 1.5 μl primer mix (5 μM forward and reverse primer) were used. The qPCR program consisted of 5 min at 95°C, followed by 40 cycles at 95°C for 3 s and 60°C for 20 s. Lastly, Ct values of technical triplicates were averaged, and fold change expression was calculated with the Delta-Delta Ct method, normalizing to GFP and HPRT1 expression.

Total RNA from different human tissues was commercially acquired from Applied Biosystems and BioChain and used as described previously (47). mRNA sequencing (mRNA-seq) was performed at Biomarker Technologies (Germany). The samples sizes for each experiment are given in the figures and legends.

### mRNA-seq analysis

Raw sequencing files (FASTQ) were adaptor and quality trimmed using trimGalore (48). Alignment of the trimmed sequencing reads to the hg19 reference genome (downloaded from Illumina’s iGenomes) was performed using Hisat2 v.2.2.1 with ‘–min-intronlen 30 –max-intronlen 3000″ parameters (49) and stored as binary alignment maps (BAM). Read count per gene tables based on BAM files were generated within R using the summarizeOverlaps function of the GenomicAlignments package (50). Normalization of read counts and detection of differentially expressed genes was calculated with DESeq2 (51) based on the read counts per gene tables. If not other indicated, significantly differentially expressed genes were chosen using a log2FC threshold of >1 or < -1 and an adjusted *P*-value < 0.05. Identification of enriched biological pathway was performed using clusterProfiler (52). Volcano plots were generated with the EnhancedVolcano R package (53).

## Results

### ZNF512B overexpression induces the formation of chromatin foci

**Figure 1.**
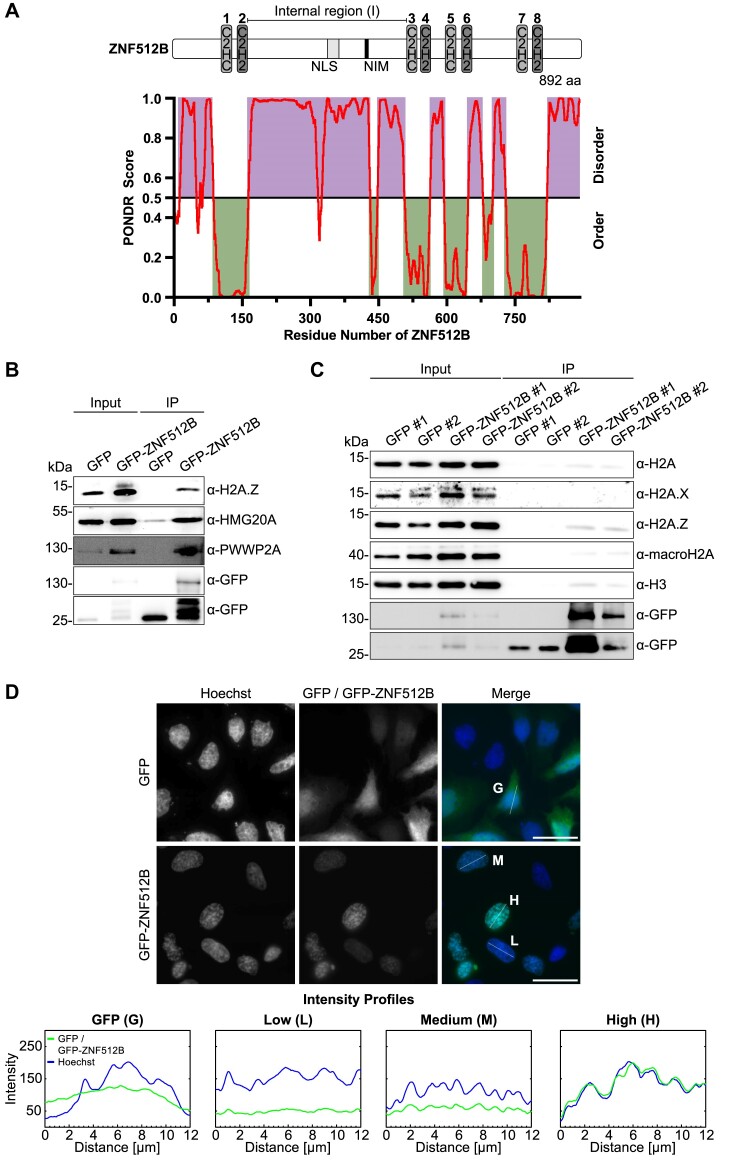
ZNF512B interacts with H2A.Z, HMG20A and PWWP2A and its overexpression leads to nuclear foci formation containing compacted chromatin. (**A**) Top: Schematic representation of human ZNF512B protein with four pairs of atypical C2HC (light gray) and typical C2H2 (dark gray) ZFs and an internal, intrinsically disordered (I) region containing an NLS (gray) and a putative NuRD interaction motif (NIM) (black). Bottom: Ordered and disordered regions of human ZNF512B (NP_065764.1) as predicted by PONDR^®^ using the predictor VLXT. (**B**) Immunoblots of nuclear extracts from HeLaK cells transiently expressing GFP or GFP-ZNF512B after pull-down with GFP-Trap beads detecting H2A.Z and its associated HMG20A and PWWP2A proteins. (**C**) Immunoblots of nuclear extracts from HeLaK cells expressing GFP or GFP-ZNF512B under a tetracycline-inducible promoter after pull-down with GFP-Trap beads detecting H2A and different H2A variants. Two clones per cell line were selected. (**D**) Top: IF microscopy of HeLaK cells expressing GFP or GFP-ZNF512B (green) co-stained with Hoechst (DNA, blue). Scale bars: 20 μm. Bottom: Intensity profiles of nuclear areas from IF pictures (see lines) depicting DNA (blue) within cells expressing GFP (G), low (L), medium (M) or high (H) levels of GFP-ZNF512B (green).

Recently, we conducted interactome studies of human H2A.Z nucleosomes, as well as of the H2A.Z-associated proteins PWWP2A and HMG20A, utilizing label-free quantitative mass spectrometry (lf-qMS) (15–17,20). One common protein identified in all screens was the ZF protein ZNF512B, which comprises eight ZF domains, arranged in four pairs. Each pair consists of one atypical C2HC followed by one typical C2H2 ZF, and the first and second pairs are separated by a large, intrinsically disordered internal region (I) housing a nuclear localization signal (NLS) (Figure 1A). ZNF512B, and especially its ZF domains, show a high level of conservation among vertebrates (Supplementary Figure S1A). Furthermore, its mRNA is expressed ubiquitously in all human tissues at low levels, with the highest content observed in brain and tumour samples (Supplementary Figure S1B).

First, we transfected HeLaK cells with plasmids encoding either GFP or a GFP-ZNF512B fusion. GFP-ZNF512B efficiently pulled down H2A.Z, PWWP2A and HMG20A, providing additional confirmation of an association between these proteins (Figure 1B). To also analyse ZNF512B’s interaction with other H2A variants, we created HeLaK cell lines expressing GFP or GFP-ZNF512B under a tetracycline-inducible promoter. GFP-ZNF512B pulled down H2A, H2A.Z and macroH2A with a visible preference for H2A.Z-containing nucleosomes (Figure 1C).

IF microscopy with transiently transfected HeLaK cells revealed that GFP-ZNF512B forms nuclear foci, whose sizes directly correlated with the level of GFP-ZNF512B expression (Figure 1D). DNA co-staining disclosed a striking co-localization of chromatin within these foci, implying that GFP-ZNF512B overexpression is associated with some form of induced DNA/chromatin compaction/condensation/aggregation. This surprising phenotype was independent of the location of the GFP-tag (Supplementary Figure S1C) and the cell type used for transfections (Supplementary Figure S1D).

Although histone post-translational modification (PTM) patterns in the nuclei were overall similar to GFP-expressing cells, a decrease in H3K4me3, H2A.Zac and H3K27ac (active histone marks) as well as a reciprocal increase in H3K27me3 and H3K9me3 (repressive histone marks) signals at the sites of aggregation was observed in a majority of cases, indicating a localization of ZNF512B to heterochromatic sites (Supplementary Figure S1E). Furthermore, GFP-ZNF512B-induced DNA/chromatin compaction foci did not represent prophase condensation spots, as the foci were not marked with the mitotic modification H3 serine 10 phosphorylation (H3S10ph) (Supplementary Figure S1F). GFP-ZNF512B chromatin foci were also distinct from senescence-associated heterochromatin foci (SAHFs) (54), as revealed by the absence of SA-β-galactosidase staining (Supplementary Figure S1G). Since it was published previously that overexpression of ZNF512B would lead to apoptosis (22), we further applied a TUNEL assay and could exclude it as underlying reason for the observed phenotype (Supplementary Figure S1H). We note that long-term expression of GFP-ZNF512B resulted in the death of cells though, which prevented us from establishing stably expressing GFP-ZNF512B cells. Due to the timing, we hypothesize that apoptosis is not actively initiated by ZNF512B but more likely a consequence of the persistent chromatin aggregation phenotype. Together, these data suggest that high levels of ZNF512B cause DNA/chromatin aggregation within the nucleus.

### Size and formation of nuclear ZNF512B/chromatin foci depend on the number of ZFs

**Figure 2.**
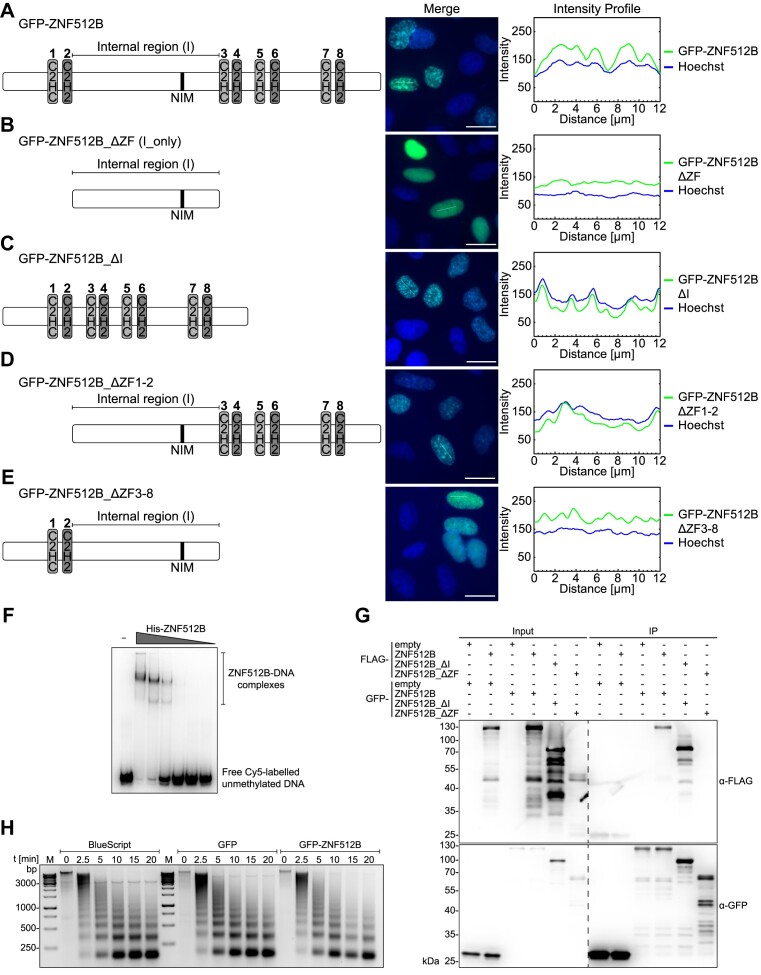
The number of ZNF512B ZFs determines extent of chromatin compaction. Left: Schematic depiction of used constructs. Middle: IF microscopy of HeLaK cells expressing (**A**) GFP-ZNF512B, (**B**) GFP-ZNF512B_ΔZF (internal domain with NIM only), (**C**) GFP-ZNF512B_ ΔI (only ZFs, without internal region), (**D**) GFP-ZNF512B_ ΔZF1-2 or (**E**) GFP-ZNF512B_ ΔZF3-8 (green) co-stained with Hoechst (DNA, blue). Scale bars: 20 μm. Right: Intensity profiles of nuclear areas from IF pictures (see lines) depicting DNA (blue)and GFP constructs (green). (**F**) EMSA of Cy5-labelled DNA together with increasing amounts of purified recombinant His-ZNF512B protein. (**G**) Immunoblots of cell extracts from HeLaK cells expressing GFP, GFP-ZNF512B, GFP-ZNF512B_ΔI or GFP-ZNF512B_ΔZF together with FLAG, FLAG-ZNF512B, FLAG-ZNF512B_ΔI or FLAG-ZNF512B_ΔZF after pull-down with GFP-Trap beads detecting GFP (IP-control) and FLAG. Dashed line indicates cut membrane. (**H**) Agarose gel of MNase (different-time points) digested chromatin isolated from HeLaK cells (control; Bluescript vector transfection; left) expressing GFP (middle) or GFP-ZNF512B (right).

Next, we set out to analyse the chromatin aggregation phenotype in more detail and determine how high levels of ZNF512B protein affect chromatin structure. We generated several GFP-ZNF512B deletion constructs, which were transiently expressed in HeLaK cells, and visualized the degree of DNA compaction by Hoechst staining. As expected, GFP-ZNF512B overexpression led to the formation of nuclear protein aggregates and large DNA foci (Figure 2A). When all ZFs were deleted and only the internal region was expressed, both protein and DNA aggregation were absent (Figure 2B), suggesting that the ZF domains are required for this phenotype. Indeed, expression of all ZFs without the internal region resulted in protein aggregation and DNA compaction/condensation similar to wild type GFP-ZNF512B (Figure 2C). Surprisingly, stepwise deletion of ZF pairs correspondingly reduced the sizes of protein aggregates and DNA foci (Figure 2D and E). As the extent of the ZNF512B and chromatin aggregation phenotype depended on the number of ZFs, we speculate that they participate in (i) DNA binding, which mediates the observed chromatin compaction/condensation, and (ii) oligomerization, explaining the protein aggregation phenotype. To first test ZNF512B’s ability to bind DNA, we performed EMSAs with purified recombinant His-ZNF512B protein (Supplementary Figure S2A). These experiments showed that ZNF512B is a DNA-binding protein (Figure 2F), independent of DNA methylation status (Supplementary Figure S2B). Moreover, co-transfections with GFP- and FLAG-tagged ZNF512B deletion constructs, followed by GFP-Trap IPs and FLAG immunoblotting, demonstrated that ZNF512B is able to form oligomers that are strictly dependent on the ZF domains (Figure 2G).

We hypothesized that ZNF512B might oligomerize and bring together chromatin domains by simultaneously binding to DNA. In such a case, ZNF512B-triggered chromatin aggregation would not result in a direct change in nucleosome occupancy but rather brings chromatin domains into closer contact. This idea is supported by a time-course MNase digestion experiment (Figure 2H), which showed no dramatic changes in chromatin accessibility after GFP-ZNF512B overexpression. These data indicate that ZNF512B binds DNA and oligomerizes, and that its ZFs are required for protein and chromatin aggregation.

### ZNF512B interacts with the NuRD complex via an internal NuRD interaction motif (NIM)

**Figure 3.**
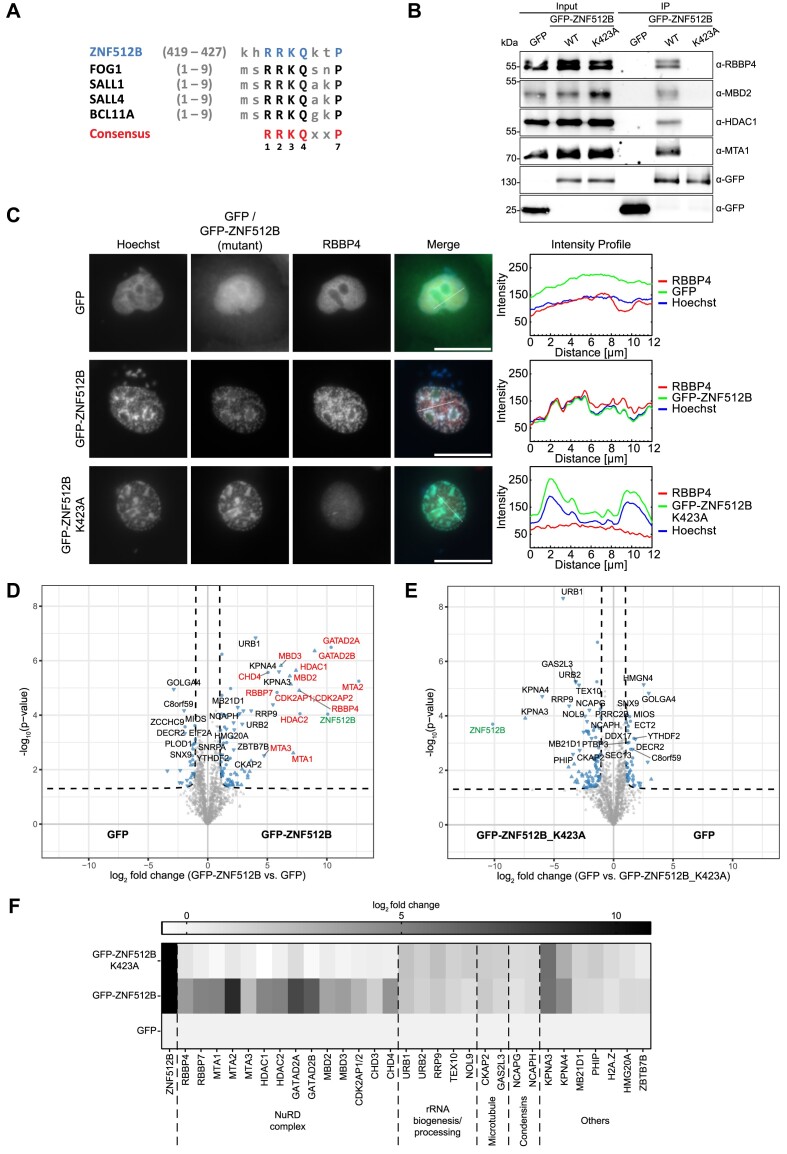
ZNF512B interacts with the complete NuRD complex via its NIM. (**A**) Alignment of ZNF512B’s putative internal NIM with N-terminal NIM sequences of other known NuRD-binding proteins. Capital letters indicate conserved amino acids. (**B**) Immunoblots of cell extracts from HeLaK cells transiently expressing GFP, GFP-ZNF512B or GFP-ZNF512B_K423A after pull-down with GFP-Trap beads detecting NuRD complex proteins. (**C**) Left: IF microscopy of HeLaK cells expressing GFP, GFP-ZNF512B or GFP-ZNF512B_K423A (green) co-stained with Hoechst (DNA, blue) and antibody against RBBP4 (red). Scale bars: 20 μm. Right: Intensity profile of nuclear areas (see lines, left) depicting DNA (blue), GFP constructs (green) and RBBP4 (red) fluorescence (see Supplementary Figure S3A and B for IF microscopy co-stains with NuRD members MTA1 and CHD4). (**D** and **E**) Volcano plots of lf-qMS data (one representative replicate) comparing proteins enriched on GFP-ZNF512B with those bound to GFP (control) (D) or comparing proteins enriched on GFP (control) with those bound to GFP-ZNF512B_K423A (E). NuRD members are highlighted in red, ZNF512B in green and other binding proteins in black. (**F**) Heatmap of label-free mass spectrometry quantification of interactors (see D and E) with either GFP-ZNF512B or GFP-ZNF512B_K423A compared to GFP. Shown is the log2-fold average enrichment of two independent experiments with four technical replicates per sample each.

In addition to its eight ZF domains, closer examination of the amino acid composition of the human ZNF512B protein revealed a sequence within its internal region (I), resembling the known NuRD interaction motif (NIM), also referred to as the FOG-1-like motif (27) (Figure 3A). This ‘RRKQxxP’ motif (where ‘x’ represents any amino acid) was initially discovered in the N-terminus of FOG-1 (27) and later found in various chromatin-regulating proteins, including Sal-like protein 1 (SALL1) (55), SALL4 (56), B-cell lymphoma/leukemia 11A (BCL11A) (57) and ZNF827 (58). This motif facilitates a direct interaction with the RBBP4 (RbAp48) subunit of the NuRD complex (38). Thus, the question arose whether ZNF512B could also interact with NuRD through this motif. To experimentally test this idea, we transiently expressed GFP alone as a control or GFP-ZNF512B in HeLaK cells and conducted IPs with GFP-Trap beads of DNase-digested cell extracts. Immunoblots of the eluted material using antibodies against several NuRD components confirmed that ZNF512B is indeed a novel NuRD-interacting protein (Figure 3B).

Next, we verified this interaction by IF microscopy. Staining of HeLaK cells expressing GFP or GFP-ZNF512B with antibodies against the NuRD-subunits RBBP4, MTA1 and CHD4 revealed a clear co-localization of these proteins at nuclear GFP-ZNF512B foci (Figure 3C; Supplementary Figure S3A and B). Having established the interaction between ZNF512B and NuRD complex members, we then investigated whether the putative NIM in ZNF512B mediates this interaction. We mutated the highly conserved lysine 423 in GFP-ZNF512B (Figure 3A) to alanine (K423A), as the corresponding residue in FOG-1 was shown to be required for NuRD binding (27). This GFP-ZNF512B_K423A mutant, when transiently expressed in the nuclei of HeLaK cells, failed to pull down NuRD proteins, in contrast to wild-type GFP-ZNF512B (Figure 3B). Accordingly, the NuRD members RBBP4, MTA1 and CHD4 did not co-localize with GFP-ZNF512B_K423A (Figure 3C; Supplementary Figure S3A and B), demonstrating that the NIM of ZNF512B is necessary for NuRD interaction. As anticipated, chromatin foci still formed when overexpressing GFP-ZNF512B_K423A. This is consistent with our previous demonstration that this DNA/chromatin compaction phenomenon is triggered even in the absence of ZNF512B’s internal domain. Nevertheless, we wondered if the loss of NuRD interaction would have any influence on the histone PTM patterns at the sites of aggregation. We repeated the corresponding IF microscopy experiments with GFP-ZNF512B_K423A expressing cells but could not observe any difference to GFP-ZNF512B expressing cells (Supplementary Figure S3C). The general level of histone PTMs in GFP, GFP-ZNF512B and GFP-ZNF512B_K423A expressing cells was likewise comparable as verified by immunoblotting (Supplementary Figure S3D). To compare the expression level of GFP-ZNF512B and GFP-ZNF512B_K423A and to show the extent of overexpression in comparison to endogenous ZNF512B, we also conducted RT-qPCR and immunoblotting (Supplementary Figure S3E and F).

We further verified the NIM-dependent NuRD interaction by performing lf-qMS after GFP-Trap IP of GFP, GFP-ZNF512B or GFP-ZNF512B_K423A. NIM-dependent binding of ZNF512B to all known NuRD complex members was observed (Figure 3D–F; Supplementary Figure S3G). Interestingly, we also detected NIM-independent binding of ZNF512B to several other proteins that are involved in nuclear processes, such as ribosome biogenesis. We biochemically confirmed the interaction of overexpressed GFP-ZNF512B and GFP-ZNF512B_K423A with Karyopherin Subunit Alpha 4 (KPNA4), a chaperone that also binds protein aggregates (59), and URB1 Ribosome Biogenesis Homolog (URB1), a protein that enables RNA binding activity (60), demonstrating the validity of our approach (Supplementary Figure S3H).

In summary, we identified a FOG-1 resembling internal NIM sequence in ZNF512B that mediates an interaction with the complete NuRD complex.

### Both ZNF512B overexpression and depletion affect gene expression in a NuRD-dependent and -independent manner

**Figure 4.**
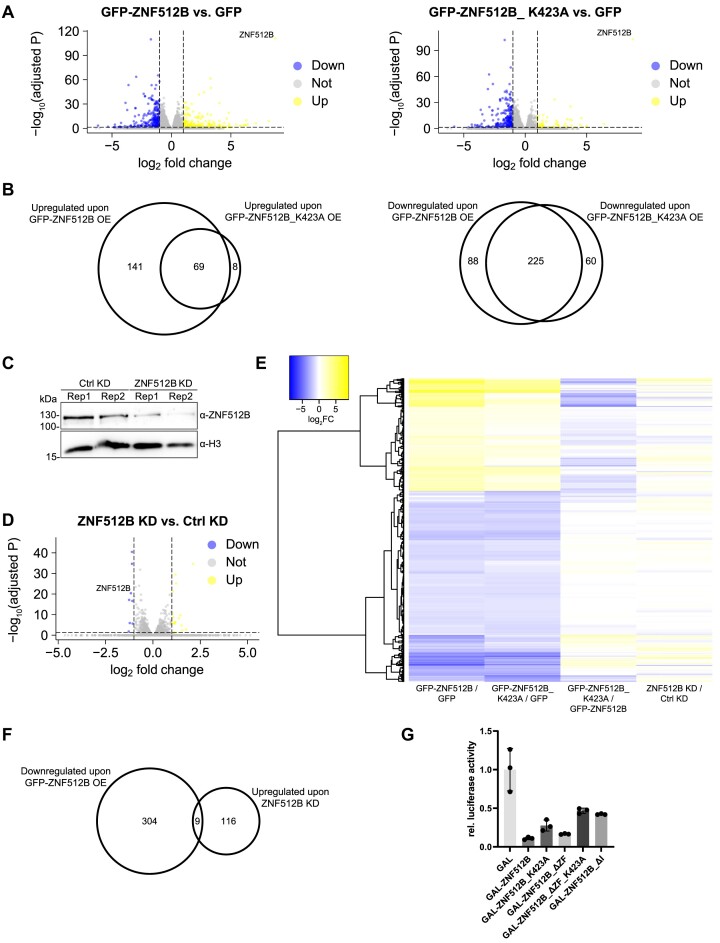
Overexpression and depletion of ZNF512B affects gene expression in NuRD-dependent and -independent manners. (**A**) Volcano plots showing significantly deregulated genes in GFP-ZNF512B (left) or GFP-ZNF512B_K423A (right) expressing HeLaK cells compared to GFP control. Depicted is log2 fold change and adjusted *P*-values. (**B**) Euler diagram depicting the overlap of significantly up- (left) or downregulated (right) genes (log2 FC > 1 or < -1 and adjusted *P*-value < 0.05) upon GFP-ZNF512B or GFP-ZNF512B_K523A overexpression compared to GFP control. (**C**) Immunoblot of nuclear extracts from two HeLaK cell replicates (Rep) two days after transfection with control (Ctrl) or ZNF512B-specific siRNA pools stained with ZNF512B or H3 (loading control) antibodies to verify ZNF512B KD efficiency. (**D**) Volcano plot showing mean of differentially deregulated genes in ZNF512B KD cells. Depicted is log2-fold change of the mean of two biological RNA-seq replicates compared to control KD. (**E**) Heatmap of deregulated genes upon GFP-ZNF512B or GFP-ZNF512B_K423A overexpression or ZNF512B depletion. Shown is the log2-fold change of GFP-ZNF512B versus GFP, GFP-ZNF512B_K423A versus GFP, GFP-ZNF512B versus GFP-ZNF512B_K423A or ZNF512B versus Ctrl KD comparisons. (**F**) Euler diagram showing the overlap of upregulated genes (log2 FC > 0.5 and adjusted *P*-value < 0.05) upon ZNF512B KD and downregulated genes (log2 FC < -1 and adjusted *P*-value < 0.05) upon GFP-ZNF512B overexpression. (**G**) Reporter assay of HEK293T cells transiently transfected with luciferase-reporter and GAL control or different GAL-ZNF512B wild-type or deletion/mutation plasmids. SD, three biological replicates.

Having demonstrated ZNF512B binding to NuRD via its NIM, we next wondered whether ZNF512B has any role in regulating gene transcription and whether such a process depends on NuRD binding. First, we transiently overexpressed GFP, GFP-ZNF512B (NuRD binding) or GFP-ZNF512B_K423A (loss of NuRD binding) in HeLaK cells, isolated RNA two days after transfection and performed high-throughput sequencing (RNA-seq) (Supplementary Figure S4A–D). We found around 500 or 350 genes to be significantly deregulated upon GFP-ZNF512B or GFP-ZNF512B_K423A overexpression in comparison to GFP expressing control cells (Figure 4A). Of those genes, 229 genes were exclusively deregulated in GFP-ZNF512B overexpressing cells, while 68 genes were exclusively deregulated upon GFP-ZNF512B_K423A overexpression (Supplementary Figure S4B).

Surprisingly, most of the commonly and GFP-ZNF512B_K423A exclusively deregulated genes were downregulated, while the majority of GFP-ZNF512B exclusively deregulated genes were upregulated (Figure 4B). Such upregulated genes were mainly expressed at extremely low levels or not expressed at all in GFP control cells (Supplementary Figure S4C). Over-representation analysis (ORA) and gene set enrichment analysis (GSEA) for both Gene Ontology (GO) and Kyoto Encyclopedia of Genes and Genome (KEGG) databases indicate that shared deregulated genes belong to several different biological processes and molecular functions, not allowing us to draw any clear biological link between ZNF512B and a precise process (Supplementary Figure S4D). These data suggest that ZNF512B is involved in transcriptional regulation, in a NuRD-dependent and -independent manner.

As we used an artificial over-expression system so far, we next wondered whether the endogenous protein also plays a role in controlling gene expression. We therefore depleted endogenous ZNF512B in HeLaK cells by siRNA-mediated KD. We observed an efficient reduction of ZNF512B mRNA and protein 2 days after transfection using RT-qPCR (Supplementary Figure S4E), immunoblots (Figure 4C) and IF microscopy (Supplementary Figure S4F). We then performed RNA-seq on these samples (Supplementary Figure S4G). We just found 18 genes to be up- and eight to be downregulated significantly upon ZNF512B depletion (Figure 4D), suggesting that endogenous ZNF512B may only have a minor effect on transcriptional regulation, at least in HeLaK cells. ORA and GSEA analyses for GO and KEGG databases revealed that deregulated genes upon depletion of ZNF512B are mainly involved in RNA-related processes (Supplementary Figure S4H). To determine direct target genes of ZNF512B that are repressed in a NuRD-dependent manner, we compared the RNA-seq data sets of all downregulated genes upon GFP-ZNF512B overexpression with upregulated genes in ZNF512B KD cells (Figure 4E). We detected only nine genes to be commonly deregulated in such a manner (Figure 4F), several of which we were also able to verify by RT-qPCR (Supplementary Figure S4I). In summary, ZNF512B is involved in the regulation of gene expression.

Lastly, we utilized a luciferase-based reporter assay in HEK293T cells to determine how promoter recruitment of ZNF512B can affect transcription directly. Interestingly, ZNF512B showed a strong repression effect (Figure 4G), suggesting that it might be involved in the inhibition rather than the activation of gene transcription. To clarify whether this effect is due to its interaction with the NuRD complex, we utilized several ZNF512B deletion or mutation constructs. The loss of NuRD binding following K423A mutation still resulted in, although reduced, over 50% repression, suggesting that NuRD interaction is only partially responsible for the inhibitory effect, as already indicated through our RNA-seq analyses. Likewise, the expression of the internal region alone without ZFs resulted in strong repression similar to the full-length ZNF512B protein, whereas the additional K423A mutation again reduced but did not completely abolish its repressor ability. Surprisingly, deletion of the entire internal region also reduced but did not eliminate the repressive effect, hinting at the existence of an additional repressive region within ZNF512B. In conclusion, ZNF512B acts as a transcriptional repressor and regulates a subset of genes in both NuRD-dependent and -independent ways.

### The ZNF512B NIM directly interacts with RBBP4

Since the association with the NuRD complex plays an important role for ZNF512B’s function as a transcriptional repressor in gene regulation, we sought to analyse this interaction in more detail. Previously, FOG-1 has been shown to bind to the RBBP4 subunit of the NuRD complex via its N-terminal NIM (38). Consequently, we investigated whether ZNF512B is also a direct interaction partner of RBBP4. We determined the X-ray crystal structure of an RBBP4–ZNF512B complex. As in previous work (38), we expressed His-tagged full-length RBBP4 in Sf9 insect cells, purified the protein, and reconstituted the RBBP4–ZNF512B complex by combining RBBP4 with a synthetic peptide comprising the ZNF512B NIM (residues 419–430). The X-ray crystal structure of this complex was determined to an overall resolution of 2.2 Å (Supplementary Data). In the asymmetric unit, two copies of the RBBP4–ZNF512B complex were found, each with essentially identical conformations. We focused on describing one of these copies.

**Figure 5.**
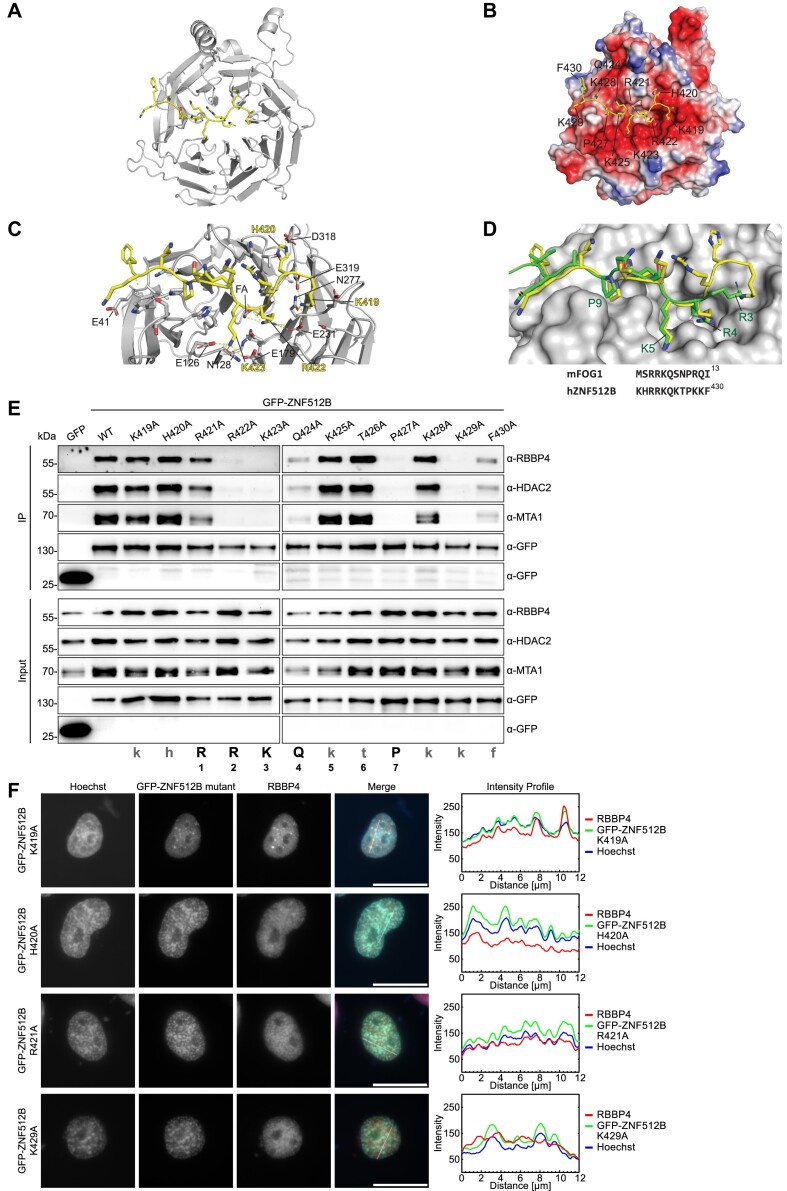
ZNF512B directly interacts with RBBP4. (**A**) Ribbon diagram of the RBBP4-ZBF512B complex (PDB: 8TX8). RBBP4 is shown in gray and ZNF512B in yellow. (**B**) Same as (A) but with RBBP4 shown as electrostatic potential surface (created in PyMOL). (**C**) Close-up of polar interactions (dashed lines) made between RBBP4 and ZNF512B. Residue numbers are indicated in black for RBBP4 and yellow for ZNF512B. (**D**) Overlay of the structure of FOG-1(1–12) (green, PDB: 2XU7) bound to RBBP4 (gray) (38) with ZNF512B (yellow, PDB: 8TX8). Key residues in FOG-1 are labeled. A sequence alignment is shown below. (**E**) Immunoblots of cell extracts from HeLaK cells transiently expressing GFP and GFP-ZNF512B NIM alanine exchange mutants after pull-down with GFP-Trap beads detecting NuRD complex members RBBP4, MTA1 and HDAC2. NIM consensus sequence is shown at the bottom. (**F**) Left: IF microscopy of HeLaK cells expressing GFP and some GFP-ZNF512B NIM alanine exchange mutants (green) co-stained with Hoechst (DNA, blue) and anti-RBBP4 antibody (red). Scale bars: 20 μm. Right: Intensity profiles of nuclear areas from IF pictures (see lines) depicting DNA (blue), GFP constructs (green) and RBBP4 (red) fluorescence. See Supplementary Figure S5B for IF of all mutants.

In the structure, RBBP4 adopts a barrel-shaped β-propeller fold with a prominent acidic cavity at one of the apical surfaces (Figure 5A and B). Residues 10–410 could be successfully modeled into the electron density map, excluding a loop encompassing residues 90–113. This loop, absent in other structures of RBBP4 (e.g. (38)), is likely to be highly dynamic; indeed, the AlphaFold2 model for human RBBP4 displays low confidence for the same region. The backbone conformation of RBBP4 in this complex also closely resembles that of the same protein in the RBBP4–FOG-1 complex (PDB: 2XU7), with a backbone RMSD of 0.29 Å (Supplementary Figure S5A).

The entire 11 residues of ZNF512B (419–430) can be confidently modeled in the structure, revealing that the peptide binds in an extended conformation to the acidic surface of RBBP4 (Figure 5B). Complex formation buries a total of 1660 Å^2^ of surface area, and a substantial number of electrostatic interactions are formed (Figure 5C). In general, residues in the C-terminal half of the ZNF512B peptide (Q424–F430) adopt very similar conformations to the corresponding residues of FOG-1 in the RBBP4–FOG-1 structure (PDB: 2XU7, (38); Figure 5D). The side chains of K425, T426 and K428 project into solvent, whereas Q424 and P427 pack into a broad uncharged groove. K429 lies on an acidic surface that is made from E41 and D74 in one of the two copies of the complex and E41 and E75 in the other. The RBBP4 backbone undergoes a localized reorientation to position either D74 or E75 near the K429 side chain.

Similarly, the side-chain positions of ZNF512B residues R422 and K423 occupy the same negatively charged pockets as the corresponding residues in FOG-1 and form similar interactions observed in FOG-1 (Figure 5B–D). Thus, K423 interacts with the side chains of E126, N128 and E179, whereas R422 forms an ion pair with E231 and the backbone carbonyl of N277. Additional electron density was observed in the R422 pocket, and this could be fitted to a molecule of formic acid, which was present in the crystallization buffer and makes electrostatic interactions with the R422 guanidinium group.

Unexpectedly, significant differences were observed in the N-terminal segment of the peptide. In the RBBP4–FOG-1 structure (Figure 5D), the side chain of R3 lies on an acidic ‘shelf’, forming electrostatic interactions with the side chains of E231, N277 and E319. However, a 130° rotation of the backbone psi angle of R421 in ZNF512B reorients the side chain of this residue, causing it to face away from RBBP4 and toward the solvent. Instead, the side-chain amino group of K419 occupies the corresponding shelf (Figure 5B) and forms an essentially identical set of interactions with RBBP4 – with the side chains of E231, N277 and E319 (Figure 5C). Furthermore, the side chain of H420 packs against the acidic pocket, interacting with D318 and contributing to this reorientation of the peptide relative to the conformation observed for FOG-1.

To independently test the requirement of each amino acid within ZNF512B’s NIM in a cellular context, we conducted an alanine mutagenesis scan. We generated a set of GFP-ZNF512B mutants, each carrying a point mutation of one residue within the NIM, and used them for GFP-Trap IPs. As expected, R422, K423 and P427 of the FOG-1-predicted ‘RRKQxxP’ motif were found to be essential for NuRD binding (Figure 5E). Similar results were obtained when examining the co-localization of all GFP-ZNF512B alanine mutants with RBBP4 in IF microscopy (Figure 5F and Supplementary Figure S5B).

Similar to a previously published FOG-1 interaction screen (27), we observed that the arginine (R421) in the first position within the consensus motif was not necessary for NuRD binding, while the glutamine (Q424) in the fourth position was only partially required (Figure 5E). Consistent with our RBBP4–ZNF512B crystal structure, we found K429 to be essential and F430 to contribute only moderately to the interaction (Figure 5E). Again, similar results were obtained when looking at the co-localization of all GFP-ZNF512B alanine mutants with RBBP4 in IF microscopy (Figure 5F and Supplementary Figure S5B). In conclusion, the NIM in ZNF512B binds to RBBP4 in a manner that is partially distinct from the mode of binding used by FOG-1 in its interaction with this NuRD component.

### Distinct amino acids in the ZNF512B NIM are required and necessary for high-affinity RBBP4 binding

**Figure 6.**
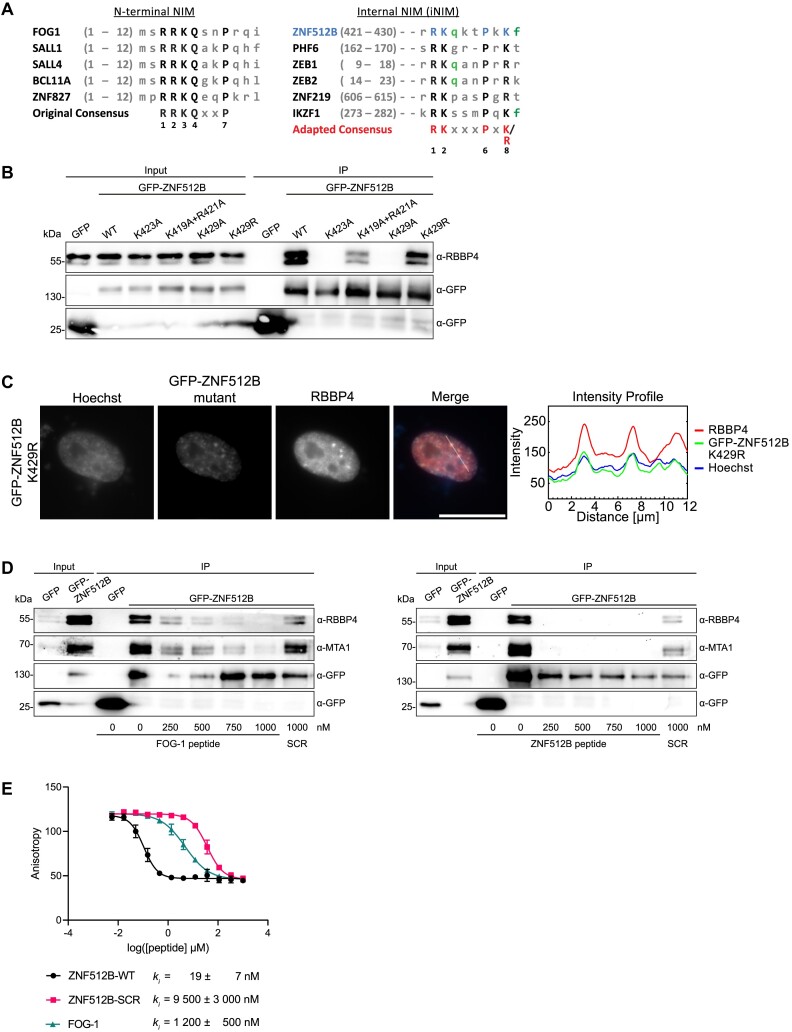
A mutational screen reveals a functional internal NIM (iNIM) sequence in ZNF512B. (**A**) Alignments of original, N-terminal (left) and internal NIM (iNIM, right) consensus sequences. Capital letters indicate conserved amino acids. Green letters indicate amino acids that contribute to, but are not essential, for RBBP4 binding. (**B**) Immunoblots of cell extracts from HeLaK cells transiently expressing GFP, GFP-ZNF512B or GFP-ZNF512B_K429R after pull-down with GFP-Trap beads detecting NuRD complex member RBBP4. (**C**) Left: IF microscopy of HeLaK cells expressing GFP-ZNF512B_K429R co-stained with Hoechst (DNA, blue) and anti-RBBP antibody (red). Scale bar: 20 μm. Right: Intensity profile of nuclear area from IF picture (see lines) depicting DNA (blue), GFP-ZNF512B_K429R (green) and RBBP4 (red) fluorescence. (**D**) Immunoblots of cell extracts from HeLaK cells transiently expressing GFP or GFP-ZNF512B mixed with increasing amounts of FOG-1 (left) or ZNF512B (right) NIM peptides or respective scrambled (SCR) controls before pull-down with GFP-Trap beads detecting NuRD complex members RBBP4 and MTA1. (**E**) FP-based binding experiment of RBBP4. Competitive experiments of unlabeled FOG-1 NIM, ZNF512B iNIM and scrambled peptides displacing the fluorescein (FAM)-labeled ZNF512B tracer (1 nM) from RBBP4 (40 nM).

Taking the data from our structure and mutagenesis screen into account, we considered whether the previously published NIM consensus sequence needs updating. We compared the known N-terminally located NIMs from FOG-1, SALL1, SALL4, BCL11 and ZNF827 (56,61–63) with the internally located sequences in PHD finger protein 6 (PHF6) (64) and ZNF512B. Based on this comparison, we propose the existence of two distinct RBBP4/NuRD interaction consensus motifs: (i) an N-terminal ‘RRKQxxP’ and (ii) an internally localized ‘RKxxxPxK’ sequence that we termed internal NIM (iNIM) (Figure 6A). Using the ScanProsite tool from Expasy, we searched for human proteins containing an iNIM and found several that have been previously connected to NuRD in some way, including Zinc finger E-box-binding homeobox 2 (ZEB2) (65) and Ikaros family zinc finger protein 1 (IKZF1) (66) (Figure 6A). Since these examples contain an arginine instead of a lysine residue at the eight position within the motif, which was shown to be necessary for NuRD interaction, we investigated whether an arginine residue at this site is also sufficient for RBBP4 interaction. We exchanged the corresponding K429 of ZNF512B for arginine and found that this mutant retains binding to NuRD, as shown by IP and IF microscopy (Figure 6B and C) experiments. Taking PHF6’s sequence into account, these data support the existence of a distinct iNIM with the consensus sequence ‘RK(x)xxPxK/R’.

Given the differences between the iNIM and NIM consensus motifs, we conducted pull-downs to investigate whether ZNF512B and FOG-1 might exhibit different binding affinities to RBBP4. Using GFP-Trap beads, we captured GFP-ZNF512B from HeLaK cell extracts in the absence or presence of increasing amounts of either FOG-1 or ZNF512B NIM peptides and detected the precipitated NuRD components RBBP4 and MTA1 in immunoblots (Figure 6D; Supplementary Figure S6A–D). Results indicated that <250 nM of the ZNF512B peptide prevented binding of GFP-ZNF512B to RBBP4 and MTA1, whereas these interactions were not completely competed with >750 nM FOG-1 peptide. This finding suggests that the iNIM in ZNF512B has a higher binding affinity for RBBP4 than the NIM in FOG-1. To quantify this observation, we applied competitive fluorescence polarization (FP)-based experiments using unlabeled ZNF512B iNIM, scrambled ZNF512B iNIM and FOG-1 NIM peptides (Supplementary Figure S6C–F) to displace a ZNF512B tracer from RBBP4. This experiment revealed a strikingly low competitive inhibition constant for ZNF512B’s iNIM (*k*_i_ = 19 ± 7 nM) in comparison to FOG-1’s NIM (*k*_i_ = 1200 ± 500 nM), indicating that ZNF512B binds RBBP4 with >60-fold higher affinity than FOG-1 (Figure 6E and Supplementary Figure S6G).In summary, we have identified a functional internal NuRD-interaction consensus motif in ZNF512B that facilitates a high-affinity binding to RBBP4.

## Discussion

We have identified ZNF512B as an RBBP4-mediated NuRD complex-interacting protein that represses transcription in both a NuRD-dependent and -independent manner. Its overexpression leads unexpectedly to protein and DNA/chromatin aggregation.

Interestingly, we observed an increase in H3K27me3 and H3K9me3 as well as a decrease in H3K4me3, H3K27ac and H2A.Zac within these aggregates, suggesting a preferential targeting of heterochromatin and the exclusion of actively transcribed euchromatic regions. During the revision process of this study, it was published by Ma *et al.* (67) that ZNF512 and ZNF512B localize to pericentric heterochromatin marked by H3K9me3, supporting our findings. We discovered that the extent of the DNA/chromatin aggregation depends on the number of ZFs present in ZNF512B. We show that ZNF512B is capable of DNA binding and oligomerization, and we propose these traits as underlying reason for the observed phenotype. Additionally, we hypothesize that this form of aggregation also promotes the interaction of ZNF512B with KPNA3/4 karyopherins. These proteins, besides their role in nuclear import (68), have been reported to act as chaperones for protein aggregation (59). While we acknowledge the non-physiological challenges associated with our artificial overexpression system, we believe it can offer new insights into ZNF512B biology. Endogenous ZNF512B mRNA is expressed ubiquitously at low levels; however, brain and certain cancer tissues exhibit elevated ZNF512B gene expression. A single-nucleotide polymorphism (SNP) located within the enhancer region of the ZNF512B gene that leads to decreased activity of the susceptibility allele in a luciferase reporter assay, correlates with ALS (26). Therefore, future experiments should investigate whether cells in these tissues or diseases also undergo changes in chromatin structures when ZNF512B protein levels are altered.

Previously, we found ZNF512B to be associated with H2A.Z and its binding partners PWWP2A and HMG20A in diverse mass spectrometry screens (15–17,32) and confirmed these associations in IP and lf-qMS experiments. We now report that ZNF512B is also a direct interactor of the NuRD complex via the RBBP4 subunit. There were previous hints about ZNF512B possibly being a binding partner of NuRD from several mass spectrometry-based surveys. These studies identified ZNF512B associated with the NuRD complex members MBD3 and GATAD2B (23,69), MTA1/2/3 (70) and HDAC1/2 but not with other HDAC family proteins (71). However, how exactly and with which NuRD complex member(s) ZNF512B interacts was not investigated. We now demonstrate that the ZNF512B–NuRD interaction is mediated by ZNF512B’s internal NIM (iNIM), facilitating a direct interaction with higher affinity than the RBBP4–FOG-1 interaction. Although we show a clear interaction between ZNF512B and RBBP4, we were unable to pull down other complexes that contain RBBP4, such as the PRC2 complex. This distinction appears to be common for proteins that bind RBBP4 via an NIM. Structural studies indicate that the targeted binding pocket for RBBP4 is blocked when the protein is engaged with PRC2 (72,73).

The internal NIM consensus sequence in ZNF512B differs from the published ‘RRKQxxP’ motif identified in the N-termini of proteins such as FOG-1 (27), SALL1 (55), SALL4 (56), BCL11A (57), ZNF296 (74) and ZNF827 (58). We found that the first arginine of the motif is dispensable for the interaction. Additionally, the conserved glutamine (Q) residue at the fourth position of the motif does not appear to be absolutely required for NuRD binding. However, interestingly, the last basic residue (eighth position) of the iNIM ‘RK(x)xxPxK/R’ sequence is essential for NuRD binding.

Through a database search using this variant NIM sequence, we identified several proteins, some of which have already been described to be associated with RBBP4 or the complete NuRD complex, such as PHF6 (64), ZNF219 (75), ZEB2 (65), Histone H3-like centromeric protein A (CENP-A) (76), IKZF1 (Ikaros) (66), IKZF2 (Helios) (77), Lethal(3)malignant brain tumor-like protein 2 (L3MBTL2) (78), T-box transcription factor 2 (TBX2) (79) and Tricho-rhino-phalangeal syndrome type I protein (TRPS1) (80). This strengthens the notion that the iNIM is a distinct RBBP4 interaction motif. However, many other proteins, such as the TF Forkhead box protein J1 (FOXJ1) or the TF Zinc finger and BTB domain-containing protein 20 (ZBTB20) (81), have not been connected to NuRD biology to our knowledge. Future experiments will provide more insights into whether these proteins are indeed novel RBBP4 and NuRD interacting proteins.

Two possible scenarios can be envisioned: (i) they may bind NuRD exclusively in a cell type-, stimulation- or differentiation-specific manner, explaining why they have not been identified in published mass spectrometry screens, and (ii) they may not bind NuRD due to other protein features surrounding the iNIM sequence, such as secondary and tertiary structures that bury the iNIM, post-translational inhibitory modifications or adjacent ‘blocking’ amino acids, or due to a competition with other interactors that prevent direct binding to RBBP4.

It is worth mentioning that NIM sequences can be further adapted. For example, the PR-SET domain-containing proteins PRDM3 and PRDM16 interact with the NuRD complex by directly binding to RBBP4 using an N-terminal ‘RxKxxxxxK’ sequence, with lysine at the third position being essential for this interaction and lysine at the ninth position forming hydrophobic and polar contacts with Q41 of RBBP4 (82). Additionally, the Epstein-Barr virus protein DNA polymerase processivity factor BMRF1 binds RBBP4 via an internal ‘RxKxxxPxK’ sequence (83). These findings suggest that a NIM sequence can be more flexible, with an amino acid inclusion between the arginine and lysine and possibly the replacement of proline.

Finally, how does ZNF512B contribute to transcriptional regulation? By binding to H2A.Z-containing chromatin regions, ZNF512B might recruit NuRD and its deacetylase and remodeling activities to regulatory genomic regions, thereby affecting gene expression. In addition, ZNF512B could also repress transcription in a NuRD-independent way, as observed in reporter assays and RNA-seq of both ZNF512B overexpressing and depleted cells. Interestingly, most differentially deregulated genes in GFP-ZNF512B overexpressing cells were upregulated. We hypothesize that GFP-ZNF512B competes with other proteins for NuRD binding and, due to its extremely high concentrations (>5000 times higher than endogenous protein, as determined by RNA-seq), binds to a large portion of the nuclear, repressive NuRD complexes, relocating them from their distinct target genes. This, in turn, could lead to an upregulation of their expression. This hypothesis is also supported by the fact that most of these upregulated genes are only expressed at extremely low levels or not at all in GFP control cells. Finally, we only find nine possible direct target genes of ZNF512B that are downregulated upon GFP-ZNF512B overexpression and upregulated upon ZNF512B depletion. Due to the small number of deregulated genes upon ZNF512B KD and the very low expression level of ZNF512B in the used HeLaK cell line, it is possible that we are not able to identify direct target genes with the approach used in this study. Nevertheless, other ZF proteins have been shown to be recruited to only one single target gene, such as ZFP568 (84), ZFP64 (85), ZNF410 (86) and ZNF558 (87). So, it is plausible to assume that ZNF512B might also have only a few or even a single target site. Future ChIP studies will provide more insights into this intriguing possibility.

In summary, we have identified ZNF512B as an RBBP4-mediated NuRD binding protein that is involved in gene repression and that causes chromatin aggregation when present at elevated levels.

## Supplementary Material

gkae926_Supplemental_Files

## Data Availability

The mass spectrometry proteomics data have been deposited to the ProteomeXchange Consortium via the PRIDE (88) partner repository with the dataset identifier PXD043366. Raw and processed RNA-seq files are deposited in GSE236639. The coordinates and structure factors of the RBBP4–ZNF512B structure have been deposited in the Protein Data Bank (PDB: 8TX8).

## Notes

Present address: Sophie E. Stebel, Institute for Biochemistry, Justus-Liebig University Giessen, Friedrichstrasse 24, 35392 Giessen, Germany.
