# Supplementary material for: ZNF512B binds RBBP4 via a variant NuRD interaction motif and aggregates chromatin in a NuRD complex-independent manner
Source: Nucleic Acids Res. 2024 Oct 26;52(21):12831–49. doi: 10.1093/nar/gkae926 (PMC11602157; doi:10.1093/nar/gkae926)
Supplement: gkae926_Supplemental_Files [file gkae926_supplemental_files.zip › Supplemental Information_Final_black.pdf]

Supplementary Information to

ZNF512B binds RBBP4 via a variant NuRD interaction motif and aggregates chromatin in a NuRD complex-independent manner

Tim Marius Wunderlich[1], Chandrika Deshpande[2,§], Lena W. Paasche[1,§], Tobias Friedrich[3,§], Felix Diegmüller[1], Elias Haddad[1], Carlotta Kreienbaum[1], Haniya Naseer[1], Sophie E. Stebel[1,&], Nadine Daus[1], Jörg Leers[1], Jie Lan[1], Van Tuan Trinh[4], Olalla Vázquez[4,5], Falk Butter[6,7], Marek Bartkuhn[3], Joel P. Mackay[2] and Sandra B. Hake[1,*]

[1]Institute for Genetics, Justus-Liebig University Giessen, 35392 Giessen, Germany
[2]School of Life and Environmental Sciences, University of Sydney, New South Wales 2006, Australia
[3]Biomedical Informatics and Systems Medicine Science Unit for Basic and Clinical Medicine, Justus-Liebig University Giessen, 35392 Giessen, Germany
[4]Department of Chemistry, Philipps University Marburg, 35043 Marburg, Germany
[5]Center for Synthetic Microbiology, Philipps University Marburg, 35043 Marburg, Germany
[6]Institute of Molecular Biology (IMB), 55128 Mainz, Germany
[7]Institute of Molecular Virology and Cell Biology, Friedrich-Loeffler-Institute, Federal Research Institute for Animal Health, 17493 Greifswald, Germany

[§]shared authors

[&]Current address: Institute of Biochemistry, Justus-Liebig University Giessen, 35392 Giessen, Germany

[*]Corresponding author: Sandra B. Hake, Institute for Genetics, Justus-Liebig-University Giessen, Heinrich-Buff-Ring 58-62, 35392 Giessen, Germany, EMAIL: sandra.hake@gen.bio.uni-giessen.de, phone: 0049 (0)641 99 35460, FAX: 0049 (0)641 99 35469

Running title: ZNF512B is a NuRD binder and chromatin aggregator

Key words: ZNF512B / H2A.Z / RBBP4 / NuRD / chromatin compaction / zinc finger

Supplementary Materials and Methods

Antibodies

Antibody	Host	Supplier	Order Number	Application	Dilution
α-CHD4	Mouse	Abcam	ab70469	IF	1:100
α-FLAG	Mouse	Sigma-Aldrich	F3165	WB	1:6,000
α-GFP	Mouse	Roche	11814460001	WB	1:3,000
α-H2A	Mouse	Active Motif	91325	WB	1:1,000
α-H2A.X	Rabbit	Sigma-Aldrich	07-627	WB	1:1,000
α-H2A.Z	Rabbit	Abcam	ab4174	WB	1:1,000
α-H2A.Zac	Rabbit	Abcam	ab232908	IF	1:100
α-H3	Rabbit	Abcam	ab1791	WB	1:5,000
α-H3K27ac	Rabbit	Active Motif	39133	IF	1:100
α-H3K27me3	Rabbit	Diagenode	C15410195	IF	1:100
α-H3K4me3	Rabbit	Diagenode	C15410003	IF	1:100
α-H3K9me3	Rabbit	Invitrogen	49-1008	IF	1:100
α-H3S10ph	Rabbit	Invitrogen	PA5-17869	IF	1:100
α-HDAC1	Rabbit	Proteintech	10197-1-AP	WB	1:1,000
α-HDAC2	Rabbit	Abcam	ab7029	WB	1:1,000
α-HMG20A	Rabbit	Proteintech	12085-1-AP	WB	1:1,000
α-KPNA4	Rabbit	Proteintech	12463-1-AP	WB	1:1,000
α-macroH2A	Rabbit	Abcam	ab13923	WB	1:1,000
α-MBD2	Rabbit	Abcam	ab188474	WB	1:1,000
α-MTA1	Rabbit	Cell Signaling Technology	5647	WB	1:1,000
				IF	1:100
α-PWWP2A	Rabbit	Norvusbio	NBP2-13833	WB	1:1,000
α-RBBP4	Rabbit	Abcam	ab79416	WB	1:1,000
				IF	1:100
α-URB1	Rabbit	Bethyl Laboratories	A305-215A	WB	1:1,000
α-ZNF512B	Rabbit	BJ-Diagnostik BioScience GmbH	not applicable	WB	1:1,000
				IF	1:100
anti-Mouse IgG (H+L), HRP	Goat	Invitrogen	31430	WB	1:20,000
anti-Rabbit IgG (H+L), HRP	Goat	Invitrogen	31460	WB	1:20,000
F(ab')2 anti-Rabbit IgG (H+L) Cross-Adsorbed, Alexa Fluor™ 488	Goat	Invitrogen	A-11070	IF	1:200

F(ab')2 anti-Rabbit IgG (H+L) Cross-Adsorbed, Alexa Fluor™ 594	Goat	Invitrogen	A-11072	IF	1:200
F(ab')2 anti-Mouse IgG (H+L) Cross-Adsorbed, Alexa Fluor™ 594	Goat	Invitrogen	A-11020	IF	1:200

Primers and Oligos

Target	Application	Forward (5' to 3')	Reverse (5' to 3')
ZNF512B	Cloning into pIRESneo-eGFP	TCATCGTTCGAAGTCCGGATCTATGACGGATCCTTTCTGCGTTGGAG	TAGTAAGCGGCCGCTCACTATCACTTTTCAGGCGCCTTGCTG
	Cloning into pEGFP-N2	ATGACGGATCCTTTCTGCGTTGGAG	CTTTTCAGGCGCCTTGCTGACTC
	Cloning into p3xFLAG-CMV-10	ATGACGGATCCTTTCTGCGTTGGAG	TCACTTTTCAGGCGCCTTGCTG
	Cloning into pFastBac1	ACTACTGAATTCATGGATTACAAGGATGACGATGACAAGGGTGGTTCTGGTACGGATCCTTTCTGCGTTGGAG	GTGGTGGGTACCTCACTTTTCAGGCGCCTTGCT
	Cloning into pAB-Gal94	TTTTGTCGACACGGATCCTTTCTGCGTTGGA	TTTTTCTAGACTACTTTTCAGGCGCCTTGCTGA
eGFP-ZNF512B	Cloning into pTetLS	TCATCGTTCGAAGCCACCATGGTGAGCAAGG	TAGTAAGTCGACTCACTATCACTTTTCAGGCGCCTTG
eGFP	Cloning into pTetLS		TAGTAAGTCGACTCACTTGTACAGCTCGTCCATGCCG
ZNF512B	Cloning of GFP-ZNF512B_ΔZF	TCATCGTTCGAAGTCCGGATCTATCAGCAGGCCGGTCACCATC	TAGTAAGCGGCCGCTCACTACTCTTCAGGGCCACCTGGAG
	Cloning of GFP-ZNF512B_ΔI fragment 1	TCATCGTTCGAAGTCCGGATCTATGACGGATCCTTTCTGCGTTGGAG	CACCTGGAGCGATGGTGACCGGCCTGCT
	Cloning of GFP-ZNF512B_ΔI fragment 2	GGTCACCATCGCTCCAGGTGGCCCTGAA	TAGTAAGCGGCCGCTCACTACACCTTCCGCTTCTTCTTGGGCTTTTCAGGCGCCTTGCTGACTC
	Cloning of GFP-ZNF512B_ΔZF1-2	TCATCGTTCGAAGTCCGGATCTATCAGCAGGCCGGTCACCATC	TAGTAAGCGGCCGCTCACTATCACTTTTCAGGCGCCTTGCTG
	Cloning of GFP-ZNF512B_ΔZF3-8	TCATCGTTCGAAGTCCGGATCTATGACGGATCCTTTCTGCGTTGGAG	TAGTAAGCGGCCGCTCACTACTCTTCAGGGCCACCTGGAG
ZNF512B	Cloning of FLAG-ZNF512B_ΔZF	ATCATCGAATTCAGCTTCGAAGTCCG	ATCATCGGTACCCTACTCTTCAGGGCC
	Cloning of FLAG-ZNF512B_ΔI	ATAACAGAATTCAATGACGGATCCTTTCTGC	AAAGCAAGATCTCTACACCTTCCGCTTCTTC

ZNF512B	Cloning of GAL-ZNF512B_ΔZF Gibson vector	CAGGTGGCCCTGAAGAGTGATTTC GGATCCAAAGCTTGATCCG	ATGGTGACCGGCCTGCTGATG AATTCCAATCTAGATTGCGGCG
	Cloning of GAL-ZNF512B_ΔZF Gibson fragment	CGCAATCTAGATTGGAATTCATCAG CAGGCCGGTCAC	ATCAAGCTTTGGATCCGAAATC ACTCTTCAGGGCCACCT
	Cloning of GAL-ZNF512B_ΔI Gibson vector	GAGTCAGCAAGGCGCCTGAATTTC GGATCCAAAGCTTGATCG	CCAACGCAGAAAGGATCCGTG AATTCCAATCTAGATTGCGGC
	Cloning of GAL-ZNF512B_ΔI Gibson fragment	CGCAATCTAGATTGGAATTCACGGA TCCTTTCTGCGTTGG	ATCAAGCTTTGGATCCGAAATT CAGGCGCCTTGCTGAC
ZNF512B	SDM K419A	GCGCACAGAAGGAAACAGAAAACA CCCAAAAAGTTTACAGGGGAGC	TTTCTGTTTCCTTCTGTGCGCT GTGCGCTCCGGGTC
	SDM H420A	AGGCCAGAAGGAAACAGAAAACAC CCAAAAAGTTTACAGGGGAGC	TTTCTGTTTCCTTCTGGCCTTT GTGCGCTCCGGGTC
	SDM R421A	AGCACGCAAGGAAACAGAAAACAC CCAAAAAGTTTACAGGGGAGC	TTTCTGTTTCCTTGCGTGCTTT GTGCGCTCCGGGTC
	SDM R422A	AGCACAGAGCGAAACAGAAAACAC CCAAAAAGTTTACAGGGGAGC	TTTCTGTTTCGCTCTGTGCTTT GTGCGCTCCGGGTC
	SDM K423A	CACAGAAGGGCACAGAAAACACCC AAAAAGTTTACAGGGGAGCAGC	GTTTTCTGTGCCCTTCTGTGCT TTGTGCGCTCCGGGTCCT
	SDM Q424A	AGCACAGAAGGAAAGCGAAAACAC CCAAAAAGTTTACAGGGGAGC	TTTCGCTTTCCTTCTGTGCTTT GTGCGCTCCGGGTC
	SDM K425A	AGCACAGAAGGAAACAGGCAACAC CCAAAAAGTTTACAGGGGAGC	TGCCTGTTTCCTTCTGTGCTTT GTGCGCTCCGGGTC
	SDM T426A	AGCACAGAAGGAAACAGAAAGCAC CCAAAAAGTTTACAGGGGAGC	TTTCTGTTTCCTTCTGTGCTTT GTGCGCTCCGGGTC
	SDM P427A	AGCACAGAAGGAAACAGAAAACAG CCAAAAAGTTTACAGGGGAGC	TTTCTGTTTCCTTCTGTGCTTT GTGCGCTCCGGGTC
	SDM K428A	AGCACAGAAGGAAACAGAAAACAC CCGCAAAGTTTACAGGGGAGC	TTTCTGTTTCCTTCTGTGCTTT GTGCGCTCCGGGTC
	SDM K429A	AGCACAGAAGGAAACAGAAAACAC CCAAAGCGTTTACAGGGGAGC	TTTCTGTTTCCTTCTGTGCTTT GTGCGCTCCGGGTC
	SDM K429R	AGCACAGAAGGAAACAGAAAACAC CCAAAAGGTTTACAGGGGAGC	TTTCTGTTTCCTTCTGTGCTTT GTGCGCTCCGGGTC
	SDM F430A	AGCACAGAAGGAAACAGAAAACAC CCAAAAAGGCTACAGGGGAGC	TTTCTGTTTCCTTCTGTGCTTT GTGCGCTCCGGGTC
	SDM K419A_R421A	GCGCACGCAAGGAAACAGAAAACA CCCAAAAAGTTTACAGGGGAGCAG	GTTTCCTTGCGTGCGCTGTGC GCTCCGGGTCC
ZNF512B	RT-qPCR	TCCCAACGACTGCTGTGAAG	TGAACTCCTTCGGACACAGC
BIRC3		CACAGAAGATGTTTCAGATCTACCA	TGTACGAACTGTACCCTTGATT GT

IL6		GCCCACCGGGAACGAAAG	CGAAGGCGCTTGTGGAG
MYRF		GCAAGTCATCGTCCGTGGTT	CACGGCAAAAGAGCCATCAGT
H19	EMSA	CACCCGGTGCTTCGGGCCCTCTAG CCCGGGCTTTTTCTAACTGGAGTG GCTCCGCCCA	

RBBP4-ZNF512B Structure: Data collection and refinement statistics

**Data collection (PDB: 8TX8)**	
Space group	P 1 21 1
Cell dimensions	
a, b, c (Å)	76.10, 59.58, 101.45
α, β, γ (°)	90, 93.77, 90
Resolution (Å)	46.88 - 2.20 (2.32 - 2.20)
R_{merge}	0.156 (1.238)
$CC_{1/2}$	0.991 (0.493)
$I / \sigma I$	6.5 (1.3)
Completeness (%)	100 (100)
Redundancy	3.5 (3.6)
**Refinement**	
Resolution (Å)	46.88 - 2.20
No. reflections	46302 (4563)
R_{work}/ R_{free}	0.21 (0.33)/ 0.24 (0.35)
Ramachandran statistics	
Favoured (%)	97.11
Allowed (%)	2.63
Outliers (%)	0.26
Number of non-hydrogen atoms	
Macromolecules	6185
Ligands	161
Solvent	337
Average *B*-factor	41.40
R.m.s deviations	
Bond lengths (Å)	0.005
Bond angles (°)	0.80

Supplementary Figure S1: Chromatin aggregation due to ZNF512B's overexpression is independent of tag localization, cell type and cell cycle stage.
(A) Alignment of ZNF512B protein sequences from *H. sapiens* (NP_065764.1), *M. musculus* (NP_001158069.1), *G. gallus* (NP_001032919.3), *X. laevis* (XP_018093756.1), *X. tropicalis* (NP_001135637.1) and *T. rubripes* (XP_029689030.1). Alignment was created in Jalview (version 2.11.3.2) using T-Coffee (version 11.00.8cbe486) with default settings. Colour indicates BLOSUM62 score. Zinc finger domains (ZF1–8) and NuRD interaction motif (NIM) are indicated by red boxes.
(B) RT-qPCR analysis of ZNF512B mRNA expression in different human cell lines and tissues normalized to HPRT1 expression. SD = three technical replicates.
(C, D) IF microscopy of Hoechst (DNA, blue) stained **(C)** HeLaK cells expressing GFP-ZNF512B or ZNF512B-GFP (green), **(D)** U2OS, HCT116 or HEK239T cells expressing GFP-ZNF512B (green). Scale bars: 20 µm.
(E, F) Left: IF microscopy of Hoechst (DNA, blue) stained HeLaK cells expressing GFP-ZNF512B (green) and co-stained with **(E)** different antibodies against active (H3K4me3, H3K27ac, H2A.Zac) or repressive (H3K9me3, H3K27me3) histone modifications (red) or **(F)** anti-H3S10ph antibody (red) as mitosis mark. Scale bars: 20 µm. **Right:** Intensity profiles of nuclear areas from IF pictures (see lines) depicting DNA (blue), GFP-ZNF512B (green) and respective histone PTMs (red) fluorescence.
(G) Bright-field microscopy of *in situ* staining for β-galactosidase activity in HeLaK cells expressing GFP or GFP-ZNF512B as marker for senescent cells. Pre-treatment of HeLaK cells with doxorubicin served as positive control. Scale bars: 20 µm.
(H) IF microscopy of TUNEL assay labelling DNA fragments with Elab Fluor® 594 (red) in DAPI (DNA, blue) stained HeLaK cells expressing GFP or GFP-ZNF512B (green). GFP expressing cells were treated with DNase I as positive control. Scale bars: 20 µm.

Supplementary Figure S2: ZNF512B overexpression does not affect global chromatin compaction.

(A) Coomassie brilliant blue-stained SDS-PAGE gel separating purified recombinant His-ZNF512B.

(B) Electrophoretic Mobility Shift Assay (EMSA) of Cy3-labelled methylated DNA together with increasing amounts of purified recombinant His-ZNF512B protein**.**

Supplementary Figure S3: ZNF512B interaction with NuRD depends on its NIM.

(A, B) Left: Immunofluorescence microscopy of HeLaK cells expressing GFP or GFP-ZNF512B_K423A (green) co-stained with Hoechst (DNA, blue) and antibody against MTA1 **(A)** or CHD4 **(B)** (red). Scale bars: 20 µm. **Right:** Intensity profiles of nuclear areas from IF pictures (see lines) depicting DNA (blue), GFP constructs (green) and MTA1 **(A)** or CHD4 **(B)** (red) fluorescence.

(C) Left: IF microscopy of Hoechst (DNA, blue) stained HeLaK cells expressing GFP-ZNF512B_K423A (green) and co-stained with different antibodies against active (H3K4me3, H3K27ac, H2A.Zac) or repressive (H3K9me3, H3K27me3) histone PTMs (red). Scale bars: 20 µm. **Right:** Intensity profiles of nuclear areas from IF pictures (see lines) depicting DNA (blue), GFP-ZNF512B_K423A (green) and respective histone PTMs (red) fluorescence.

(D) Immunoblots of DNase-digested nuclear extracts from HeLaK cells expressing GFP, GFP-ZNF512B or GFP-ZNF512B_K423A detecting active (H3K4me3, H2A.Zac, H3K27ac) or repressive (H3K27me3, H3K9me3) histone PTMs and H3 (loading control).

(E) RT-qPCR to detect relative ZNF512B mRNA expression upon GFP, GFP-ZNF512B or GFP-ZNF512B_K423A overexpression in HeLaK cells normalized to HPRT1 expression. SD = three biological replicates.

(F) Immunoblots of nuclear extracts from HeLaK cells expressing GFP, GFP-ZNF512B or GFP-ZNF512B_K423A detecting ZNF512B and GFP.

(G) Volcano plot of lf-qMS data (one representative replicate) comparing proteins enriched on GFP-ZNF512B with those bound to GFP-ZNF512B_K423A. NuRD members are highlighted in red and other binding proteins in black. See also Figure 3F for heatmap.

(H) Immunoblots of nuclear extracts from HeLaK cells transiently expressing GFP, GFP-ZNF512B or GFP-ZNF512B_K423A after pull-down with GFP-Trap beads detecting NIM-independent binding of KPNA4 and URB1. *: cut membrane.

Supplementary Figure S4: Gene expression changes upon GFP-ZNF512B or GFP-ZNF512B_K423A overexpression or ZNF512B depletion.

(A) Principal component analysis (PCA) of RNA-seq data depicting three replicates of GFP (red), GFP-ZNF512B (green) or GFP-ZNF512B_K423A (blue) expressing HeLaK cells.

(B) Euler diagram depicting the overlap of significantly deregulated genes (log2 FC > 1 or < -1 and adjusted p-value < 0.05) upon GFP-ZNF512B or GFP-ZNF512B_K523A overexpression compared to GFP control.

(C) Box plot depicting gene expression base levels (RPKM) of HeLaK cells upon GFP overexpression. Shown are genes significantly deregulated upon GFP-ZNF512B overexpression divided in upregulation or downregulation.

(D) Gene set enrichment analysis (GSEA) for the Gene Ontology (GO) database of deregulated genes upon GFP-ZNF512B and GFP-ZNF512B_K423A overexpression.

(E) RT-qPCR to detect relative ZNF512B mRNA expression upon control (Ctrl) or ZNF512B siRNA-mediated knock-down (KD) in HeLaK cells normalized to HPRT1 expression. SD = three biological replicates.

(F) IF microscopy of HeLaK cells upon control (Ctrl) or ZNF512B siRNA-mediated knock-down (KD) co-stained with anti-ZNF512B antibody (green) and Hoechst (DNA, blue). Scale bars: 20 µm.

(G) Principal component analysis (PCA) of RNA-seq data depicting two replicates of control (Ctrl) or ZNF512B siRNA-mediated KD in HeLaK cells.

(H) Gene set enrichment analysis (GSEA) for the Gene Ontology (GO) database of deregulated genes upon ZNF512B knock-down (KD).

(I) RT-qPCR to detect relative BIRC3, IL6 or MYRF mRNA expression upon GFP, GFP-ZNF512B or GFP-ZNF512B_K423A overexpression (top) or control (Ctrl) or ZNF512B siRNA-mediated knock-down (KD) (bottom) in HeLaK cells normalized to HPRT1 expression. SD = three biological replicates. RNA-seq data: BIRC3 and IL6 genes were downregulated in GFP-ZNF512B overexpression and upregulated upon ZNF512B KD; MYRF was upregulated upon GFP-ZNF512B overexpression.

Supplementary Figure S5. **Binding of the ZNF512B NIM to RBBP4.**

(A) Overlay (over backbone heavy atoms) of the RBBP4-ZNF512B structure (grey and yellow, PDB: 8TX8) with the RBBP4-FOG-1 structure (salmon and green, PDB: 2XU7). **(B) Left:** IF microscopy of HeLaK cells expressing GFP-ZNF512B NIM alanine mutants co-stained with Hoechst (DNA, blue) and anti-RBBP antibody (red). Scale bars: 20 µm. **Right:** Intensity profiles of nuclear areas from IF pictures (see lines) depicting DNA (blue), GFP-ZNF512B mutants (green) and RBBP4 (red) fluorescence.

Supplementary Figure S6: ZNF512B contains a functional internal NIM.

(A–F) Characterization of peptides used in peptide competition and fluorescence polarization (FP) experiments. Peptides were synthesized in 5 µmol scale. **(A)** HPLC chromatogram of purified peptide FOG1-WT. Gradient 5-95% MeCN monitored at 220 nm. Sequence: H_2N-MSRRKQSNPRQIKRS-Ahx-Ahx-K-$CONH_2$. After purification (5-50% MeCN), the 8 x TFA salt product (2.23 mg, 0.71 µmol, 14% yield) was obtained as a white solid. t_R = 6.56 min. Purity ≥ 99%. Formula: $C_{93}H_{173}N_{37}O_{24}S$. Molecular weight: 2224.32 Da. HRMS-ESI+ (m/z): $[M+3H]^{3+}$ calcd.: 742.7806; found: 742.7887. **(B)** HPLC chromatogram of purified peptide FOG1-SCR. Gradient 5-95% MeCN monitored at 220 nm. Sequence: H_2N-RRSIQKMRQPKNSRS-Ahx-Ahx-K-$CONH_2$. After purification (5-50% MeCN), the 8 x TFA salt product (2.22 mg, 0.71 µmol, 14% yield) was obtained as a white solid. t_R = 6.68 min. Purity ≥ 99%. Formula: $C_{93}H_{173}N_{37}O_{24}S$. Molecular weight: 2224.32 Da. HRMS-ESI+ (m/z): $[M+3H]^{3+}$ calcd.: 742.7807; found: 742.7876. **(C)** HPLC chromatogram of purified peptide ZNF512-WT. Gradient 5-95% MeCN monitored at 220 nm. Sequence: H_2N-KHRRKQKTPKKFTGE-$CONH_2$. After purification (5-50% MeCN), the 9 x TFA salt product (3.06 mg, 1.06 µmol, 21% yield) was obtained as a white solid. t_R = 6.47 min. Purity ≥ 99%. Formula: $C_{82}H_{142}N_{30}O_{20}$. Molecular weight: 1868.19 Da. HRMS-ESI+ (m/z): $[M+3H]^{3+}$ calcd.: 624.0429; found: 624.0427. **(D)** HPLC chromatogram of purified peptide ZNF512B-SCR. Gradient 5-95% MeCN monitored at 220 nm. Sequence: H_2N-KQHGKTEKTPRKRFK-$CONH_2$. After purification (5-50% MeCN), the 9 x TFA salt product (2.10 mg, 0.72 µmol, 14% yield) was obtained as a white solid. t_R = 6.12 min. Purity ≥ 99%. Formula: $C_{82}H_{142}N_{30}O_{20}$. Molecular weight: 1868.19 Da. HRMS-ESI+ (m/z): $[M+3H]^{3+}$ calcd.: 624.0429; found: 624.0431. **(E)** HPLC chromatogram of purified peptide ZNF512B-FAM. Gradient 5-95% MeCN monitored at 220 nm. Sequence: H_2N-KHRRKQKTPKKFTGE-Ahx-Ahx-K(FAM)-$CONH_2$. After purification (5-50% MeCN), the 9 x TFA salt product (7.47 mg, 2.07 µmol, 41% yield) was obtained as a white solid. t_R = 9.43 min. Purity ≥ 98%. Formula: $C_{121}H_{186}N_{34}O_{29}$. Molecular weight: 2580.98 Da. HRMS-ESI+ (m/z): $[M+1H]^{1+}$ calcd.: 1291.2150; found: 1291.2137. **(F)** HPLC chromatogram of purified peptide FOG1. Gradient 5-95% MeCN monitored at 220 nm. Sequence: H_2N-MSRRKQSNPRQI-$CONH_2$. After purification (5-50% MeCN), the 5 x TFA salt product (3.49 mg, 1.67 µmol, 34% yield) was obtained as a white solid. t_R = 6.23 min. Purity ≥ 99%. Formula: $C_{60}H_{110}N_{26}O_{17}S_1$. Molecular

weight: 1499.7443 Da. HRMS-ESI+ (m/z): $[M+2H]^{2+}$ calcd.: 750.4204; found: 740.4191.

(G) FP-based saturation binding experiment of RBBP4 to 1 nM fluorescein (FAM)-labelled ZNF512B in 50 mM Tris-HCl, 150 mM NaCl, 0.02% Triton™ X-100.
