# Supplementary figures and images for: ZNF512B binds RBBP4 via a variant NuRD interaction motif and aggregates chromatin in a NuRD complex-independent manner
Source: Nucleic Acids Res. 2024 Oct 26;52(21):12831–49. doi: 10.1093/nar/gkae926 (PMC11602157; doi:10.1093/nar/gkae926)

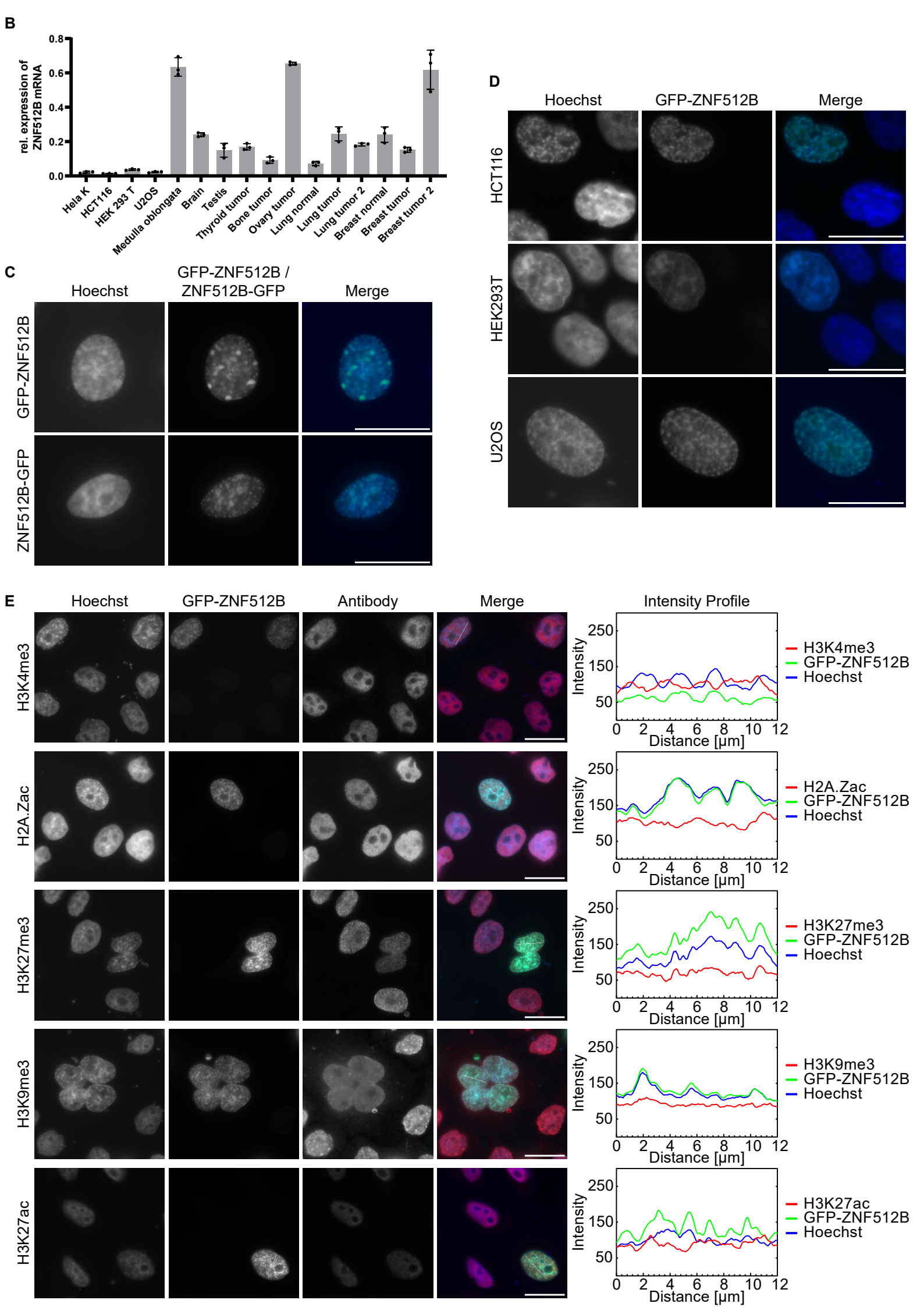

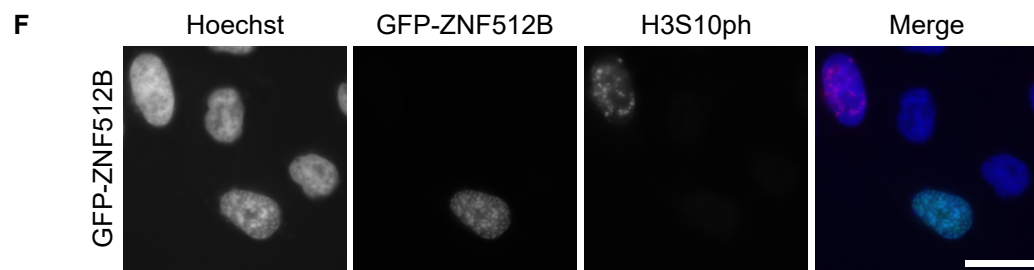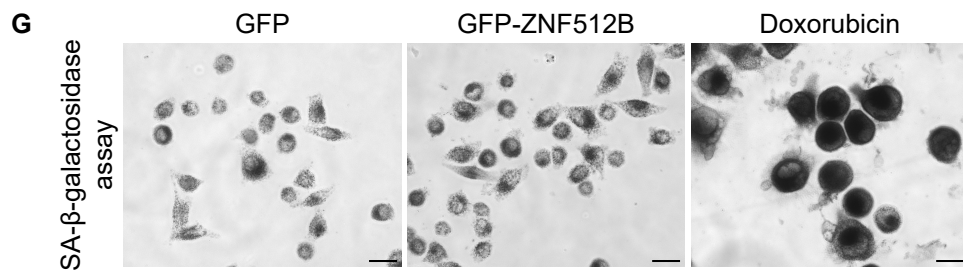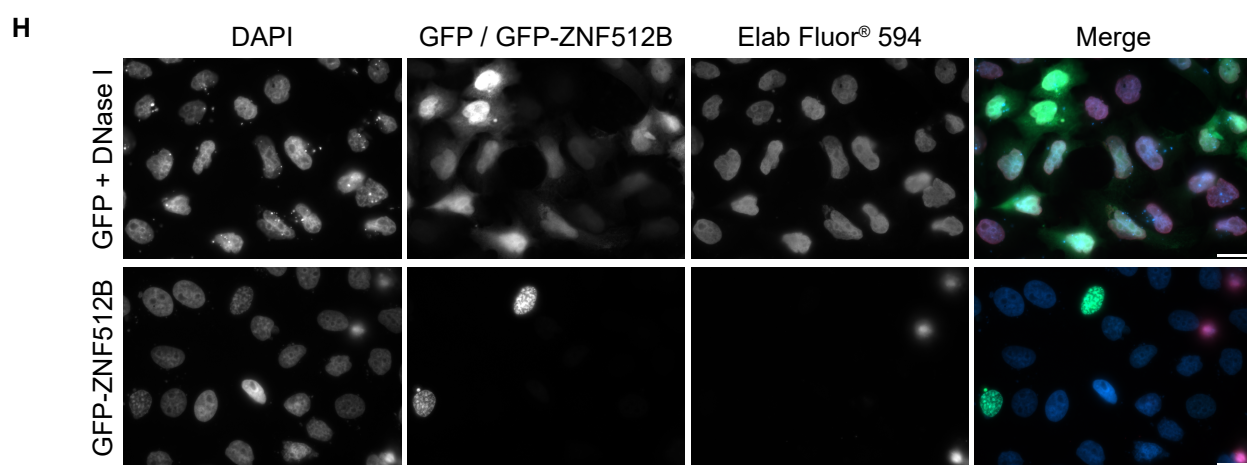

Supplement: gkae926_Supplemental_Files [file gkae926_supplemental_files.zip › Figure S1 Kopie.pdf]

**A**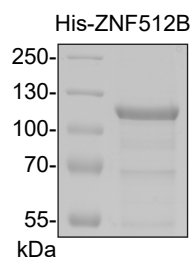**B**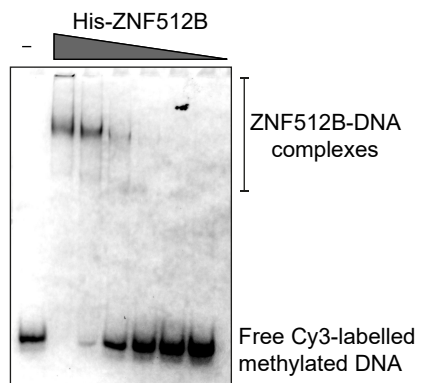

Supplement: gkae926_Supplemental_Files [file gkae926_supplemental_files.zip › Figure S2 Kopie.pdf]

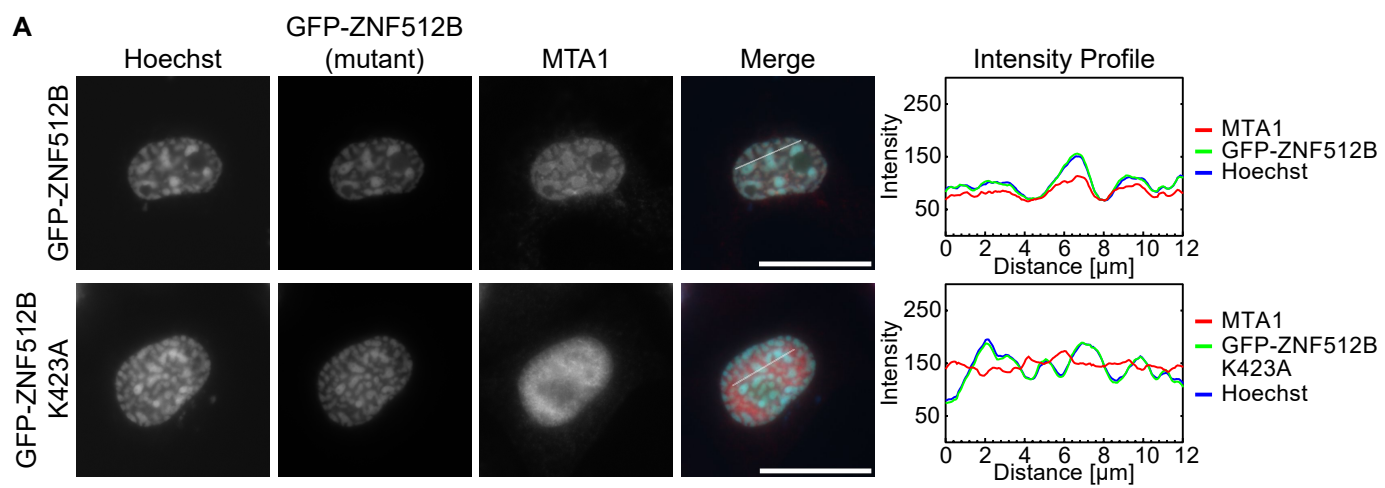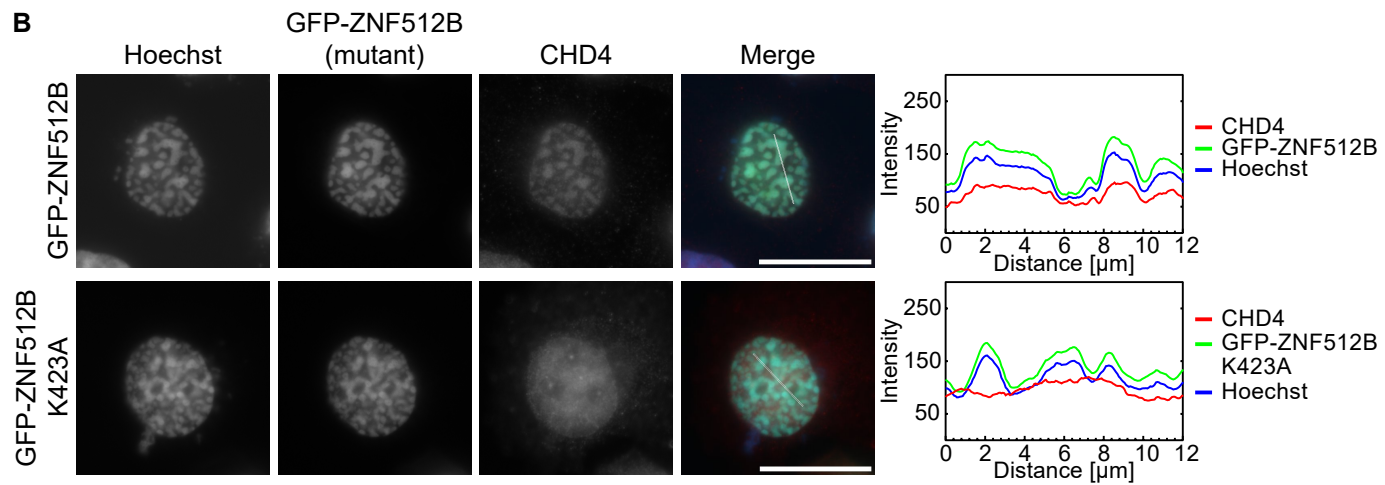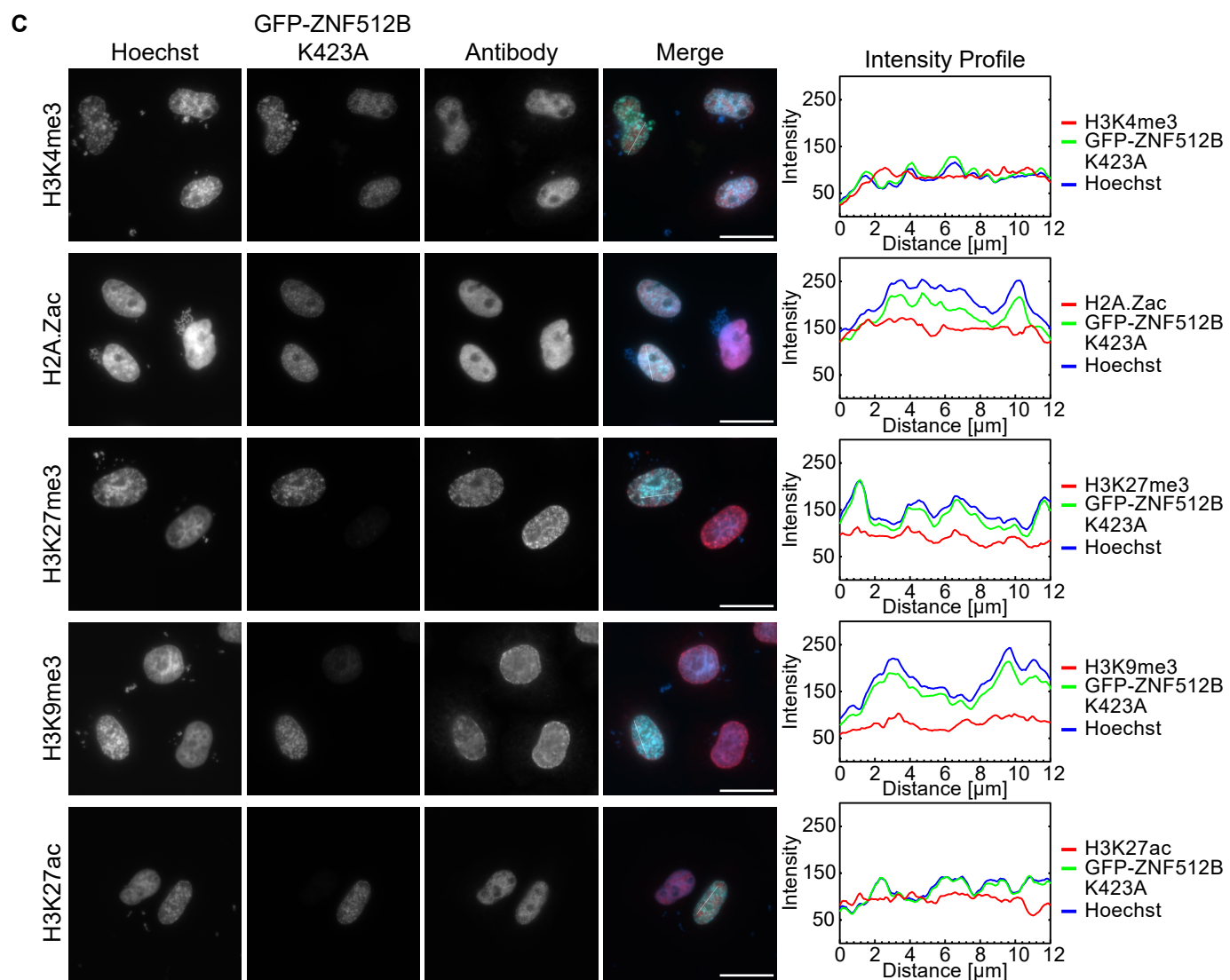

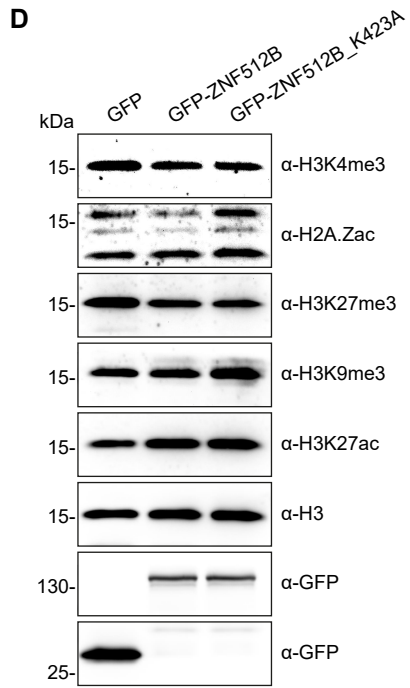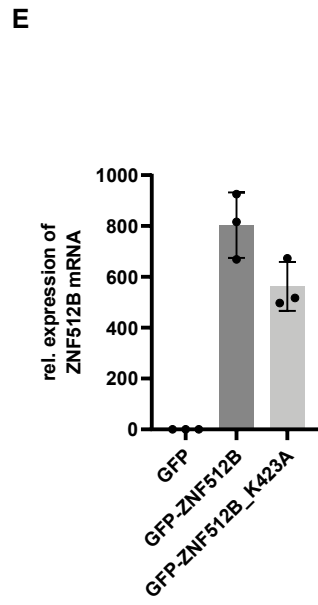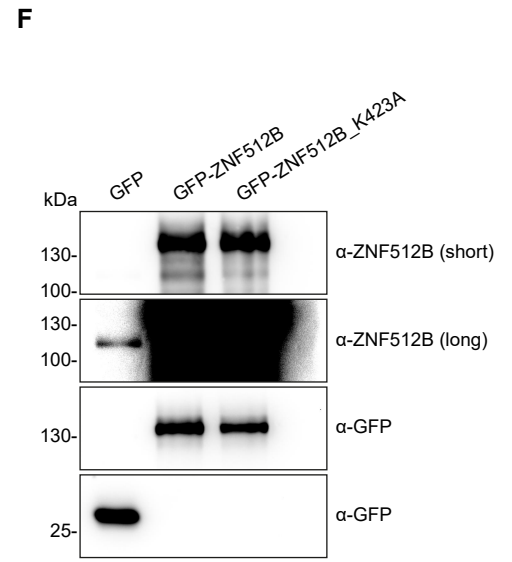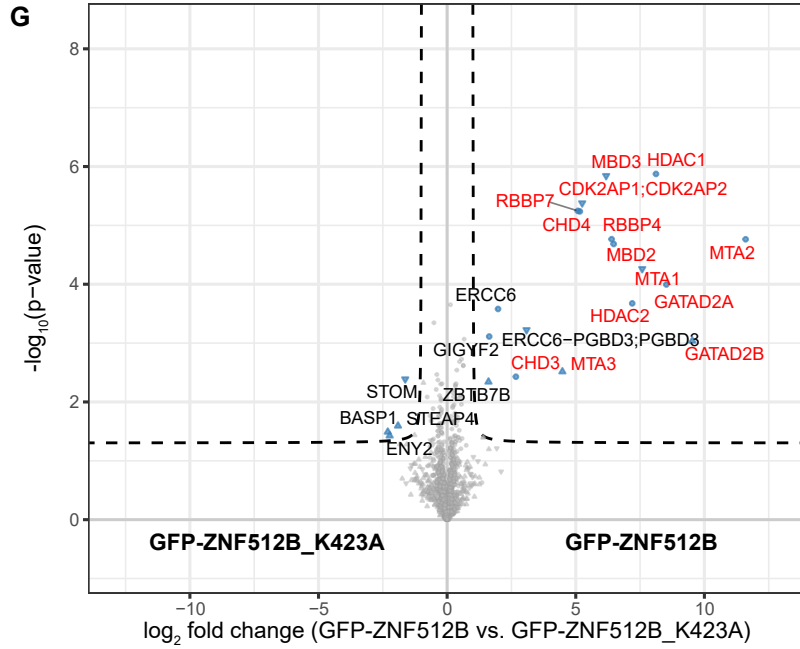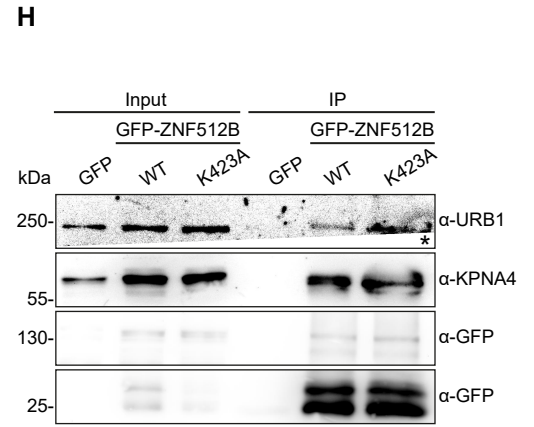

Supplement: gkae926_Supplemental_Files [file gkae926_supplemental_files.zip › Figure S3 Kopie.pdf]

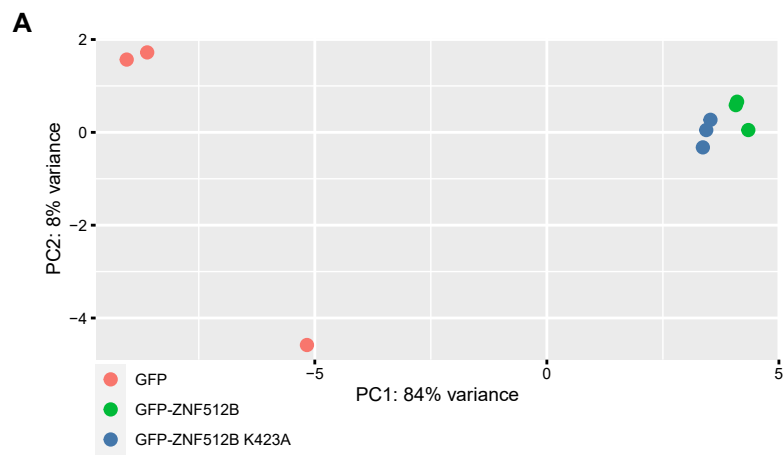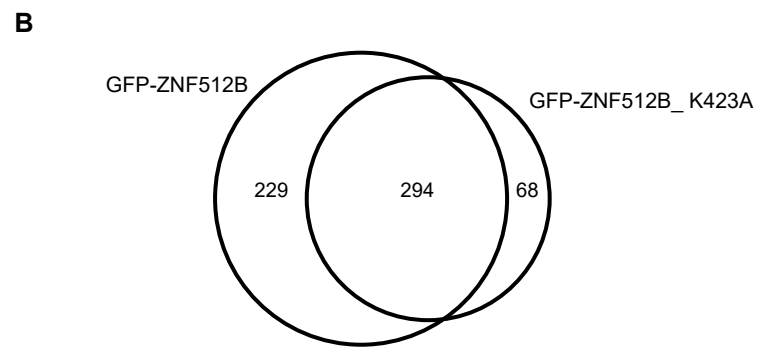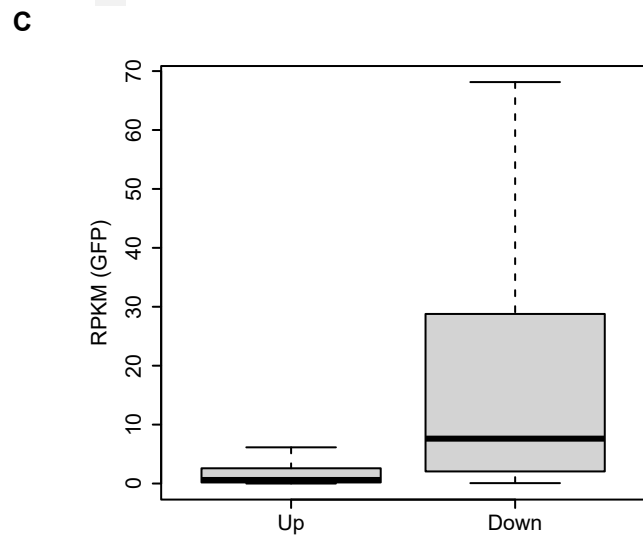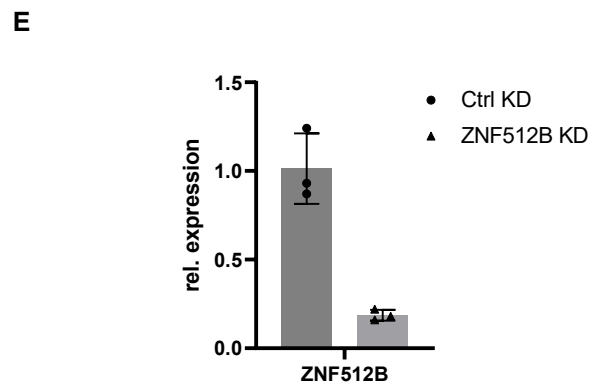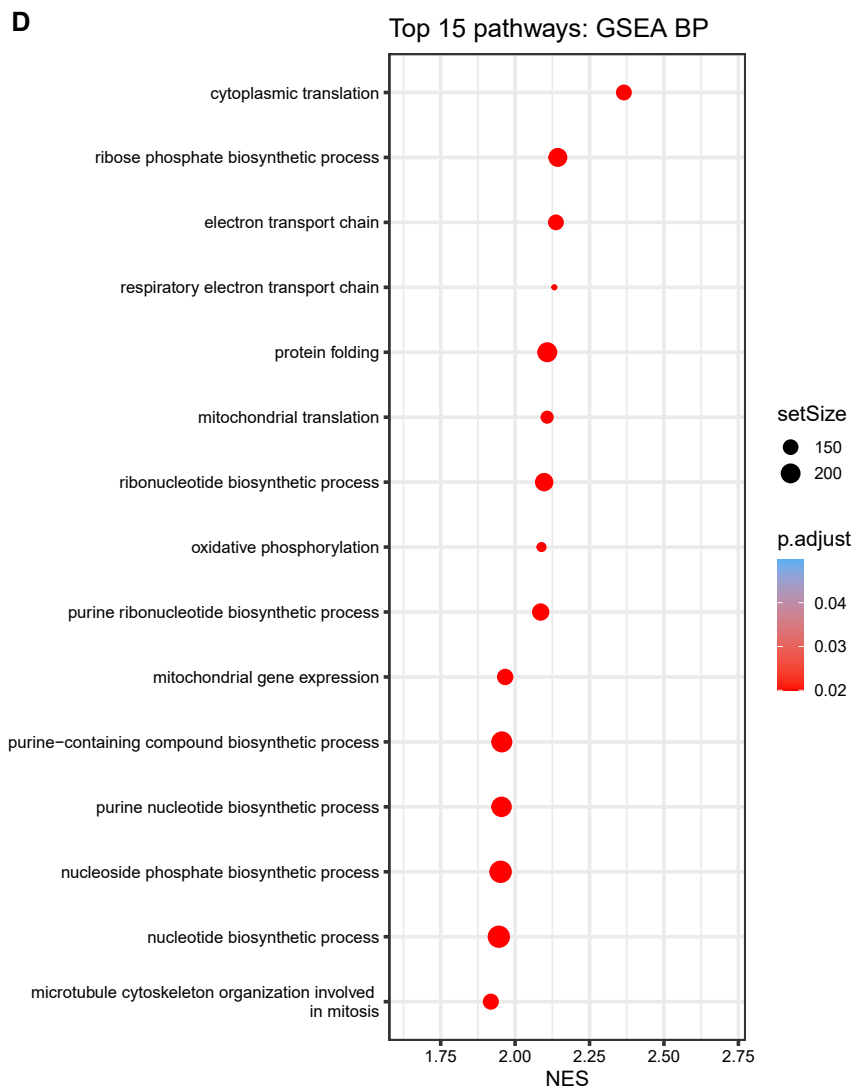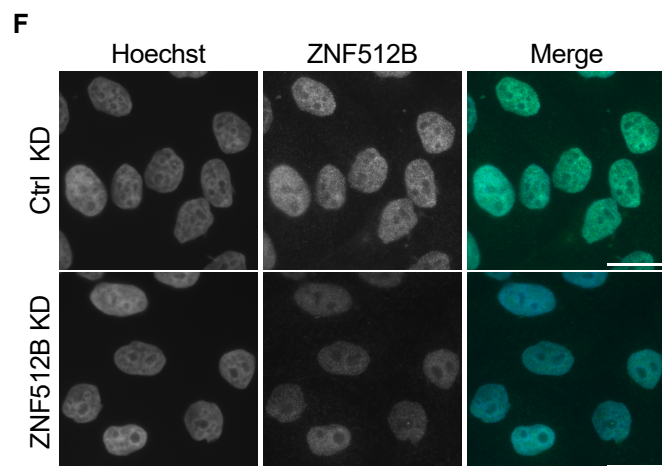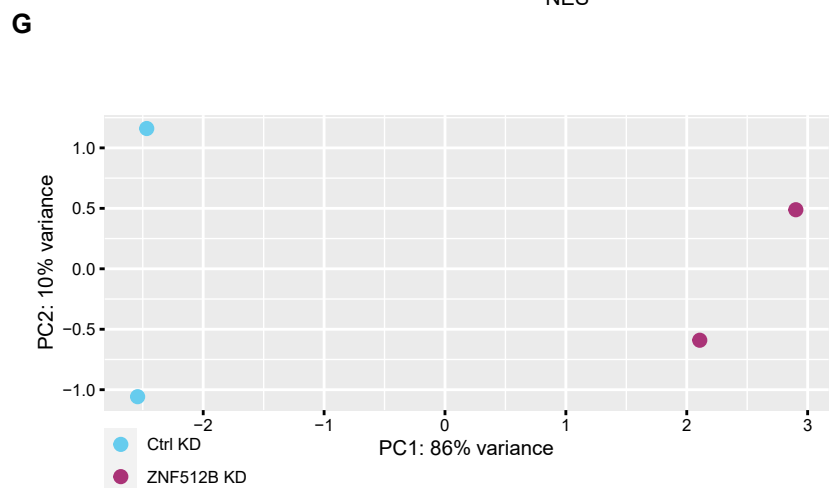

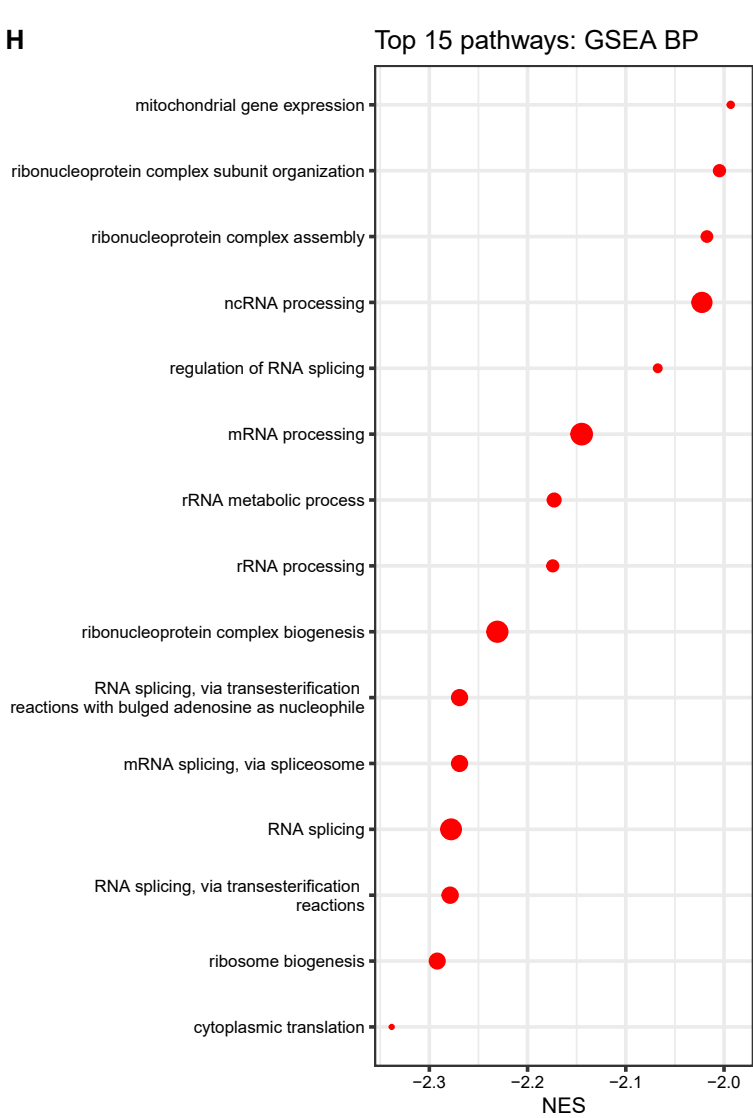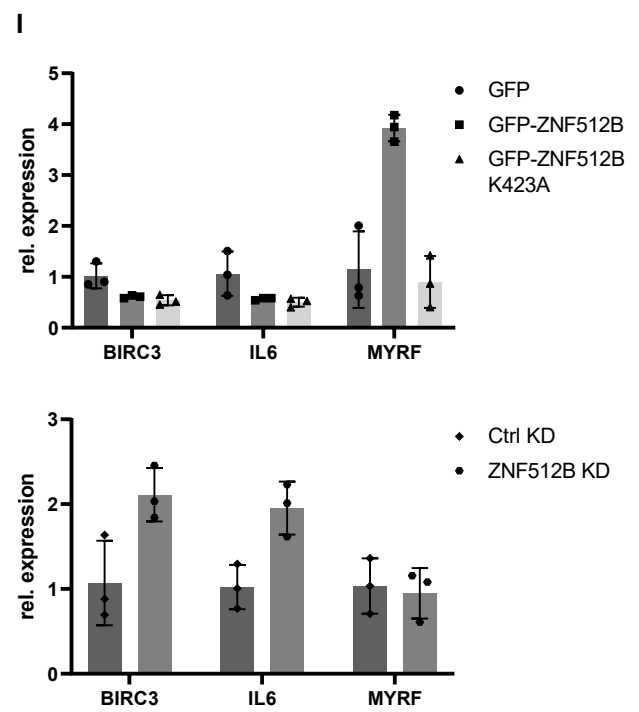

Supplement: gkae926_Supplemental_Files [file gkae926_supplemental_files.zip › Figure S4 Kopie.pdf]

**A**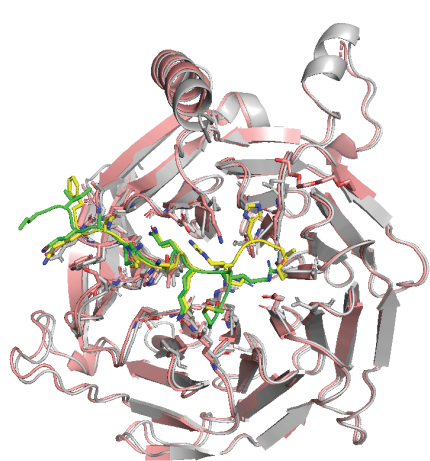**B**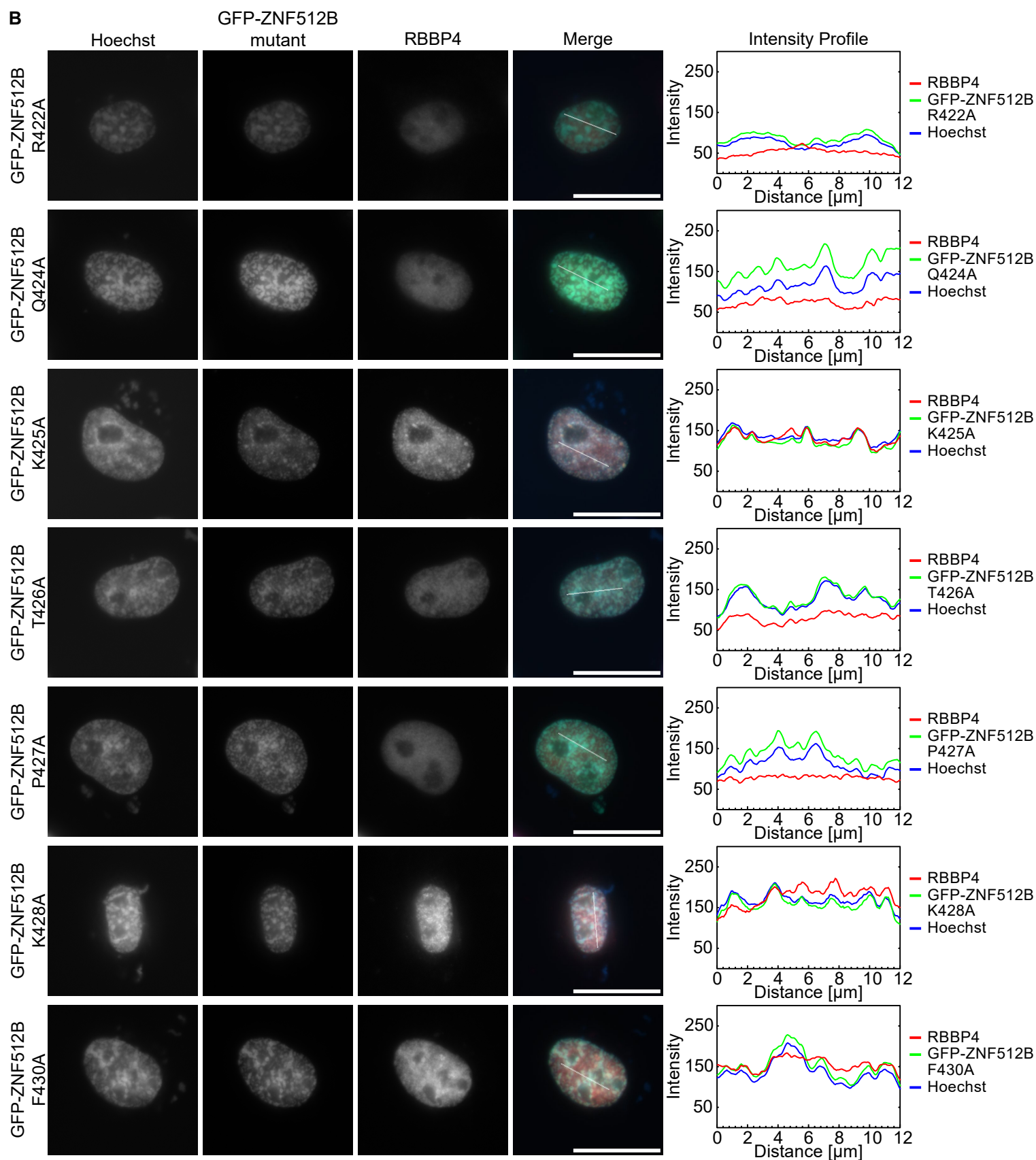

Supplement: gkae926_Supplemental_Files [file gkae926_supplemental_files.zip › Figure S5 Kopie.pdf]

**A**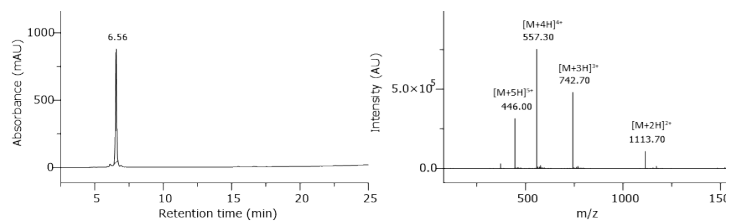**B**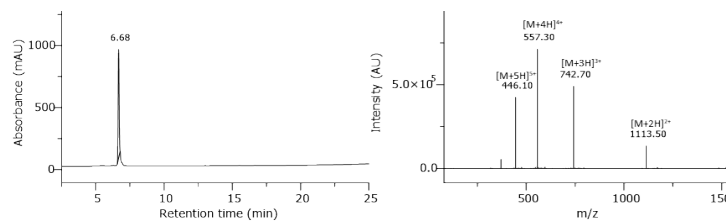**C**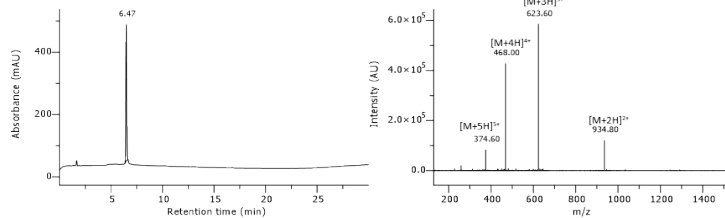**D**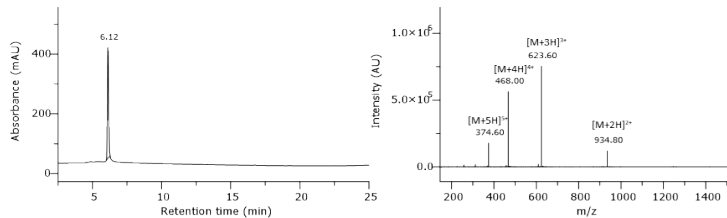**E**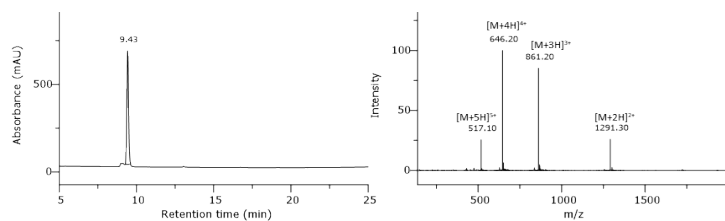**F**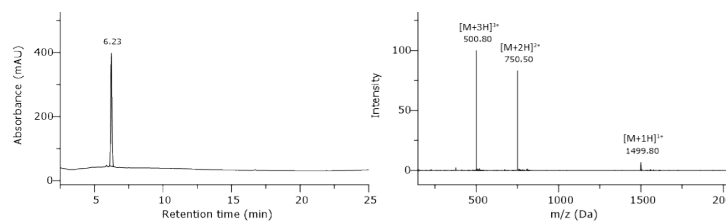**G**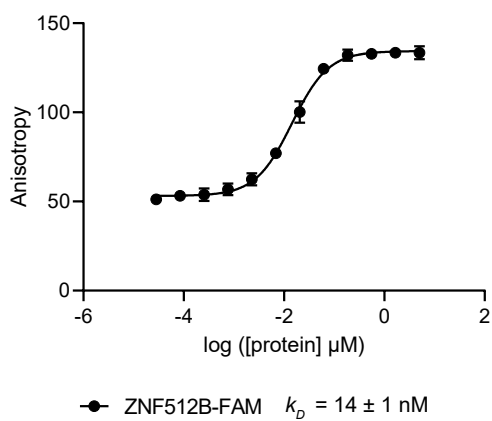

Supplement: gkae926_Supplemental_Files [file gkae926_supplemental_files.zip › Figure S6 Kopie.pdf]
